# A Molecular Clock Infers Heterogeneous Tissue Age Among Patients with Barrett’s Esophagus

**DOI:** 10.1371/journal.pcbi.1004919

**Published:** 2016-05-11

**Authors:** Kit Curtius, Chao-Jen Wong, William D. Hazelton, Andrew M. Kaz, Amitabh Chak, Joseph E. Willis, William M. Grady, E. Georg Luebeck

**Affiliations:** 1 Division of Gastroenterology, University of Washington School of Medicine, Seattle, Washington, United States of America; 2 Program in Computational Biology, Fred Hutchinson Cancer Research Center, Seattle, Washington, United States of America; 3 Clinical Research Division, Fred Hutchinson Cancer Research Center, Seattle, Washington, United States of America; 4 Gastroenterology Section, VA Puget Sound Health Care System, Seattle, Washington, United States of America; 5 University Hospitals Case Medical Center, Case Western Reserve University School of Medicine, Cleveland, Ohio, United States of America; Johns Hopkins University, UNITED STATES

## Abstract

Biomarkers that drift differentially with age between normal and premalignant tissues, such as Barrett’s esophagus (BE), have the potential to improve the assessment of a patient’s cancer risk by providing quantitative information about how long a patient has lived with the precursor (i.e., dwell time). In the case of BE, which is a metaplastic precursor to esophageal adenocarcinoma (EAC), such biomarkers would be particularly useful because EAC risk may change with BE dwell time and it is generally not known how long a patient has lived with BE when a patient is first diagnosed with this condition. In this study we first describe a statistical analysis of DNA methylation data (both cross-sectional and longitudinal) derived from tissue samples from 50 BE patients to identify and validate a set of 67 CpG dinucleotides in 51 CpG islands that undergo age-related methylomic drift. Next, we describe how this information can be used to estimate a patient’s BE dwell time. We introduce a Bayesian model that incorporates longitudinal methylomic drift rates, patient age, and methylation data from individually paired BE and normal squamous tissue samples to estimate patient-specific BE onset times. Our application of the model to 30 sporadic BE patients’ methylomic profiles first exposes a wide heterogeneity in patient-specific BE onset times. Furthermore, independent application of this method to a cohort of 22 familial BE (FBE) patients reveals significantly earlier mean BE onset times. Our analysis supports the conjecture that differential methylomic drift occurs in BE (relative to normal squamous tissue) and hence allows quantitative estimation of the time that a BE patient has lived with BE.

## Author Summary

Barrett’s Esophagus (BE) is a metaplastic precursor to esophageal adenocarcinoma (EAC). When a patient is diagnosed with BE, it is generally not known how long he/she has had this condition because BE is asymptomatic. While the question of how long a premalignant tissue or lesion has been resident in an organ (dwell time) may not be of importance for cases where curative interventions are readily available (such as adenomas in the colon), for BE, curative interventions are either costly or carry patient risks. Knowledge of a precursor’s dwell time may therefore be advantageous in determining the cancer risk due to the stepwise accumulation of critical mutations in the precursor. In this study, we create a molecular clock model that infers patient-specific BE onsets from DNA methylation data. We show that there is considerable variation in the predicted BE onset times which translates, using mathematical modeling of EAC, into large variation in individual EAC risks. We make the case that, notwithstanding other known risk factors such as chronological age, gender, reflux status, etc., knowledge of biological tissue age can provide valuable patient-specific risk information when a patient is first diagnosed with BE.

## Introduction

There is great interest in the molecular characterization of precancerous fields and lesions (e.g., colorectal adenomas or ductal carcinoma in situ (DCIS) in the breast) to quantify their neoplastic potential, although it is generally not known how long such lesions (or fields) have sojourned in a patient when they are discovered. This point is of particular importance in the case of Barrett’s esophagus (BE), a variable-length metaplastic precursor of esophageal adenocarcinoma (EAC) that has been shown to undergo a stepwise progression to cancer involving multiple rate-limiting events [1–3]. In spite of a generally low EAC progression risk of about 0.2–0.5% per year across BE patients [4], the progression risk is believed to be highly variable and dependent on age, gender, histopathological grade, and personal risk factors such as severity of gastroesophageal reflux disease (GERD), body mass index (BMI), and smoking status [5]. However, since the total number of BE patients who progress to EAC is generally low for most epidemiological studies (mostly due to limited follow-up), inter-individual variability in progression risk is difficult to specify other than by gross factors. Furthermore, the clinical assessment of the BE tissue is known to be fraught with uncertainty as only a small portion of the tissue is biopsied for pathology. Thus, there is a pressing need to develop more accurate markers (and risk stratifications) that identify BE that is more likely to progress to EAC in a person’s lifetime versus BE that is indolent or has low neoplastic potential.

Inter-individual variability in the EAC progression risk may depend on the duration of how long a patient has lived with BE (BE dwell time). In a large population-based study in Northern Ireland, Bhat et al. [6] found a significant increase of the annual progression risk with patient age (2-fold from age <50 to age 60–69) suggesting that the BE-to-EAC progression risk is not constant but rather increases with the age of the BE tissue due to the stepwise accumulation of genetic and epigenetic alterations that drive premalignant and malignant progressions in BE [1, 2, 7]. Thus, a longer dwell time for BE may increase the risk for neoplasia and cancer in an exponential manner consistent with the exponential increases observed in the age-specific incidence of EAC in the general population [8, 9]. Also, in an environment of chronic inflammation analogous to that which is caused by GERD within BE, patients with ulcerative colitis have a higher colon cancer risk that increases with earlier age of onset and disease duration [10, 11]. These risk factors unfortunately cannot be identified clinically in the case of BE because BE is asymptomatic. Yet, the use of mathematical modeling to quantifiy the waiting (or dwell) time of premalignant stages during carcinogenesis until the occurrence of cancer has been of considerable interest [12].

Recently identified age-related changes in DNA-methylation have led to the notion of a biological tissue age which, although highly correlated with chronological age, may differ significantly from it [13, 14]. It is generally believed that epigenetic drift (i.e., neutral changes in DNA methylation levels) is responsible for this process [15]. In this study we examine array-based methylation patterns of CpG-dinucleotides across the genome to determine whether CpGs that drift differentially between BE and normal tissue can be used to infer the relative biological age of a patient’s BE tissue. Specifically, we identify CpGs that undergo such ‘methylomic drift’ based on array data from formalin fixed paraffin embedded (FFPE) tissue samples from two groups of BE patients: one group of 10 patients each with 2 or more tissue samples that were obtained at least 5 years apart (data set D1). These samples provide longitudinal information at the individual level. A second group of 30 patients ranging in age from 21 to 88 (data set D2) had matched tissue samples obtained from Barrett’s esophagus and adjacent normal esophagus squamous epithelium (SQ), providing cross-sectional information as well as differential drift information between SQ and BE tissue. The combined statistical analyses of these two data sets, as described in Materials and Methods, suggest that numerous hypomethylated CpG sites undergo significant differential methylomic drift in BE versus SQ. Significantly, the observed patient-specific drift differentials appear relatively uniform across the set of identified 67 CpGs, giving rise to high correlations in the methylation differentials (against the mean drift) between CpGs. Thus, a hallmark of methylomic drift is that the associated methylation differentials between markers (across patients) are highly correlated, as are all clocks that keep time. We also validated the computed methylomic drift rates for the 67 selected CpGs in an independent data set of 10 additional BE patients (data set DV) each with samples at two time points.

To infer patient-specific BE onset times from the measured methylation levels of identified CpGs that drift differentially between BE and SQ tissues, we use a Bayesian model that accounts for (CpG-specific) random effects in drift rates, measurement error, and a patient-specific BE onset time. Furthermore, to gain insights into how the age of BE onset may influence EAC risk, we used a recently developed mathematical model for EAC incidence to compute standardized lifetime risks for the individuals in data set D2 given their predicted BE onset times [8, 16]. Additionally, we applied this methodology to methylation array data from 22 familial BE (FBE) patients (data set D3). The quantitative predictions of both BE onset times and inferred EAC risks for BE patients without neoplasia (D2) and familial BE (D3) suggest that *BE onset* is a useful event-marker of cancer risk. In the following we describe the data and methodologies that support this conclusion.

## Materials and Methods

All CpG-methylation data for this study were generated with the Infinium HumanMethylation450 beadchip arrays (Illumina) [17, 18] that include over 485,000 CpG-methylation sites throughout the genome (covering 99% of Reference Sequence (RefSeq) genes (National Center for Biotechnology Information (NCBI), Bethesda, MD, USA). Data normalization was performed using the R Bioconductor *minfi* package, which includes background level corrections, color adjustments and Subset-quantile Within Array Normalization (SWAN) normalization. SWAN is specifically designed for HumanMethylation450 array data to account for systemic differences between the Infinium I and Infinium II probe designs [19]. Next we filtered out unreliable, gender bias, and noisy probes from downstream analysis, including probes having the average detection p-values across samples greater than 0.05, chromosome X-associated probes, and those containing at least one SNP with low minor allele frequency (MAF = 0) in the probe body [20, 21]. For linear regressions of the probe-specific methylation fractions on patient age we used M-values rather than *β*-values to better account for epigenetic drift that occurs at very low (<1%) and high levels of methylation. M-values are logit_2_-transformed *β*-values (computed using Illumina’s formula *β* = *M*/(*M* + *U* + 100)), allowing for non-linear saturation effects of methylation fractions with age at both ends of the methylation spectrum. Note, at the molecular level, CpG-methylation is essentially a binary variable (a CpG dinucleotide is either methylated or unmethylated). However, in a tissue sample, only cell population averages can be measured across all epigenomes in that sample.

### Ethics statement

The human tissues used for the analyses presented here were obtained from 72 patients with confirmed Barrett’s esophagus (BE). Written informed consent was obtained, signed by all participants, and conformed to institutional ethics requirements. IRB approval (protocol numbers 1989, 8137) was given by the ethical review board of the Fred Hutchinson Cancer Research Center.

### Patient data

**Fig 1:**
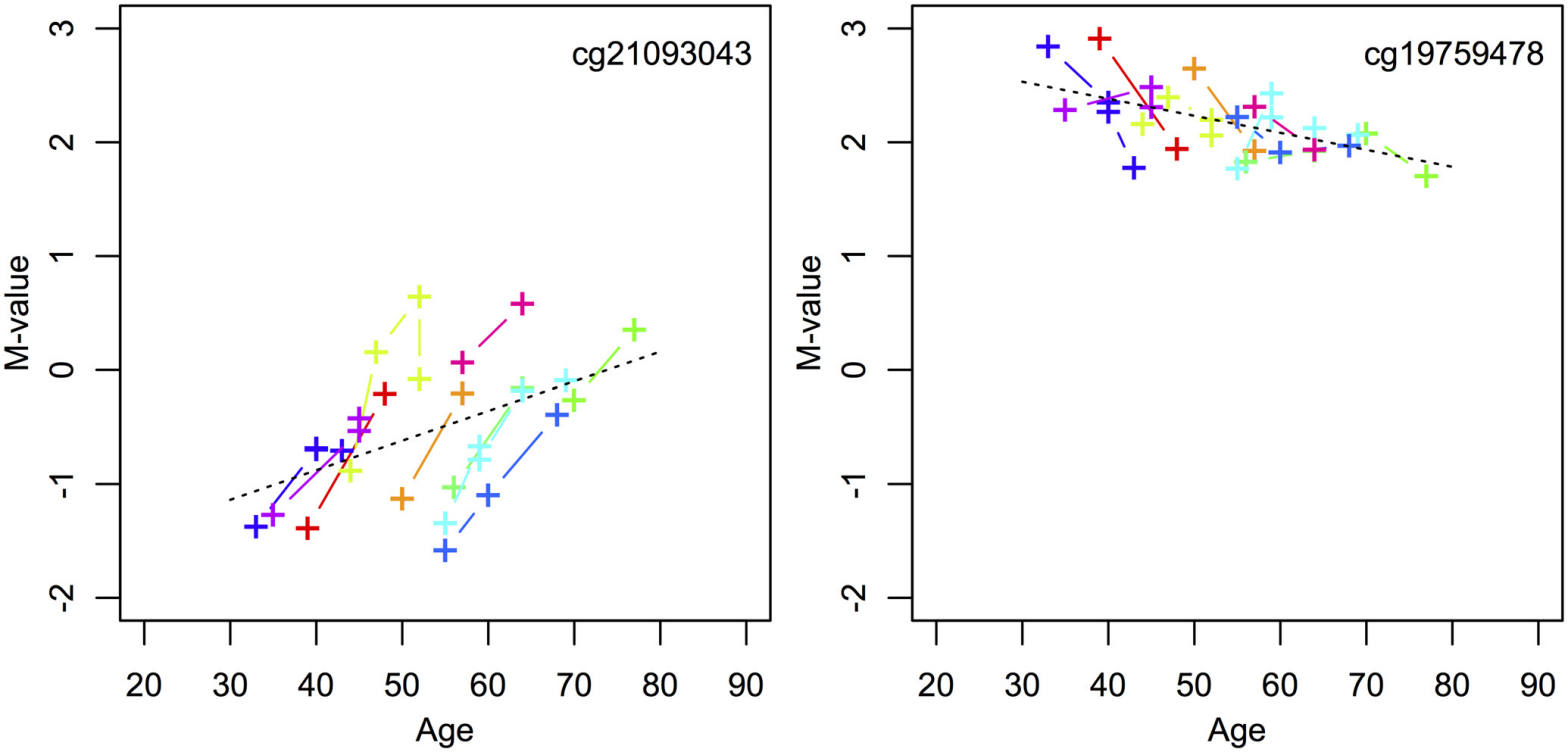
Longitudinal drift from set D1. Examples of one CpG (cg21093043) that significantly drifts up (left panel, becomes increasingly hypermethylated) and one CpG (cg19759478) that significantly drifts down (right panel, becomes increasingly hypomethylated) among longitudinal data points (See Step 1 of Material and Methods). Each individual from data set D1 provides serial samples denoted by color. To illustrate significant population drift across all serial samples for these two CpGs, the black dotted lines show the aggregate regression lines across all samples for cg21093043 (p-value = .005) and cg19759478 (p-value = .001).

We examined levels of DNA methylation at over 450,000 CpG sites in tissue samples from four groups of BE patients (see S1 Table for detailed patient information). The first data set (D1) is unique and consists of serial samples from 10 BE patients, ages 33–70 years at index biopsy (mean age = 51.2), with 2 or more tissue biopsies each that were collected at least 5 years apart to comprise a total of 29 samples. D1 patient data for two particular CpGs that show longitudinal drift for each of these 10 patients’ serial sample sets are shown in Fig 1.

**Fig 2:**
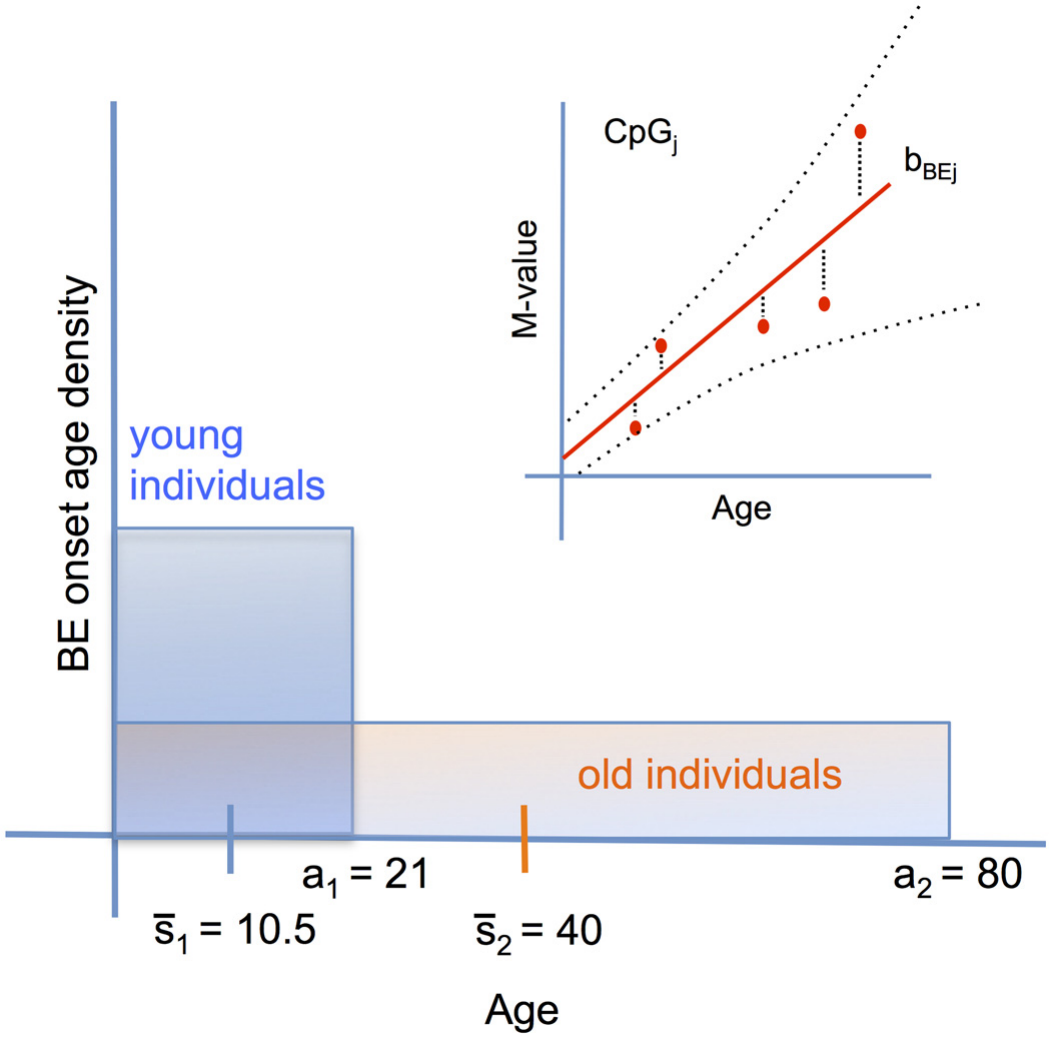
Population drift from set D2. The heterogeneity with age around the mean population drift rate may be caused by the inter-individual heterogeneity of BE onset times from a tissue of origin. Illustration of cross-sectional BE data for a certain CpG_j_ is shown in upper right inset with mean population rate *b*_*BEj*_. Due to lack of data on onset age, we assume *a priori* uniform, flat distributions for BE onset times. Thus, two example patients from data set D2, who had biopsies taken at index endoscopy ages *a*_1_ = 21 and *a*_2_ = 80, would have mean BE onset times ${\bar{s}}_{1}=a_{1}/2=10.5$ and ${\bar{s}}_{2}=a_{2}/2=40$, respectively. Older patients diagnosed with BE are expected to show greater mean and variance in BE onset ages compared to younger patients.

The second, cross-sectional data set (D2) includes matched BE and normal squamous esophageal epithelium (SQ) tissue samples from 30 BE patients ages 21–88 years (mean age = 63.4) comprising a total of 60 tissue samples. While the D1 data provide some information on methylomic drift in BE tissue for each patient, the aggregated cross-sectional data also provide population-level information on the mean drift rate across all patients and ages. Although methylomic drift may depend on various factors, here we will focus on the influence of BE dwell time, which may be highly variable from patient to patient, even for patients of similar age. Fig 2 shows the probability densities of BE onset for two representative D2 patients’ ages at time of biopsy (*a*_1_ = 21, *a*_2_ = 80), and the theoretical consequence their ages will have on the statistical inference of their BE onset ages. The inter-individual heterogeneity in BE onset times will thus affect the methylation level data around the mean population drift. An illustration for a single CpG site *j* for the BE samples from D2 is shown in the insert of Fig 2. Note, for the cross-sectional group (D2), the matched BE and SQ samples originate from biopsies collected during the same endoscopic exam.

The third serial data set (DV) consists of 10 BE patients from Cleveland Clinic Foundation, ages 54–77 years at index biopsy (mean age = 51.2), with 2 serial tissue biopsies each, comprising a total of 20 BE samples.

The fourth data set (D3) includes BE tissue samples from 22 familial BE (FBE) patients ages 39–84 years (mean age = 62.8) with one sample per patient. Familial Barrett’s esophagus (FBE) was defined as having a first- or second-degree relative with long-segment BE, adenocarcinoma of the esophagus, or adenocarcinoma of the gastroesophageal junction whose diagnosis was confirmed by review of endoscopy and histology reports [22]. The data also include gender and age when the tissue biopsy was collected for each patient (see S1 Table).

### Identification of markers of differential methylomic drift

Two concepts have so far emerged that relate alterations in DNA methylation to biological tissue age. The first is based on the discovery of sets of *clock-CpGs* that undergo age-dependent changes in methylation that in combination correlate strongly with chronological age [13, 14, 23]. The second concept relates to subtle changes in methylation levels due to epigenetic drift as a result of a semi-conserved replication process of DNA-methylation patterns [24–27]. Significantly, some CpG-islands that show very low (hypo-)methylation levels early in life are known to undergo gradual methylation over time, presumably as a result of sporadic *de novo* methylation events during DNA replication, a process commonly understood as *epigenetic* or *methylomic drift* [15, 24, 28–31]. Therefore, to narrow the number of CpG candidates that may serve as markers for differential tissue aging in the emerging metaplastic tissue of BE patients, we first identified CpGs that show significant longitudinal drift among the patients of our longitudinal study D1, as described below.

The following steps summarize our discovery pipeline in more detail.

#### Step 1: Identify BE *drift-CpGs* using longitudinal data

To identify CpGs that show consistent drift across all patients in D1, we examined the relationship between incremental changes in methylation levels (M-value) and time since first biopsy for all D1 patients as shown in Fig 1. For marker *j* and individuals (*i* = 1, …, 10) each with longitudinal samples obtained at times *t*_*ik*_, where *k* enumerates the individual-level samples, we fitted linear regression models for incremental M-value changes in marker *j* as a function of time since each individual’s first biopsy. Specifically, we model Δ*M*_*ijk*_ = *b*_*j*_(*t*_*ik*_ − *t*_*i*1_) + *ϵ*_*ijk*_ across all individuals in set D1, in aggregate, with biopsy collection at times *t*_*ik*_. We thus identified candidate CpGs that undergo concordant incremental drift across these patients and determined the drift rates, *b*_*j*_, *j* = 1, .., *M* (regression slopes) for all available markers in the batch. We applied a highly permissive false discovery rate (FDR) of *q* = 0.20 for the incremental drift analysis to avoid excessive pruning of potentially informative candidates. To ensure that these CpGs that appear to drift incrementally (with time-since-first-biopsy) also drift cross-sectionally with age, we simultaneously tested each CpG for concomitant cross-sectional drift across all samples in D1 together using a nominal p-value = 0.01 (illustrated by black dotted lines in Fig 1). Ultimately, this testing identified 2,950 CpGs out of a total of 456,579 CpGs that drift upward and 1,781 CpGs that drift downward across the 10 D1 patients. While the ‘in-aggregate’ regressions for incremental and population drift clearly ignore the inter-individual variability in the estimated drift rates, *b*_*j*_, *j* = 1, .., *M* for the *M* candidates we were able to identify, there appears to be some heterogeneity in the drift rates between markers. However, for simplicity and because of the relatively small number of samples available in D1, we assume homogeneity of the associated drift rate distributions, i.e., the drift rates are assumed to have prior distributions of the form of single (positively or negatively centered) normal distributions during Bayesian inference.

#### Step 2: Identify SQ vs BE differential drift in cross-sectional data

**Fig 3:**
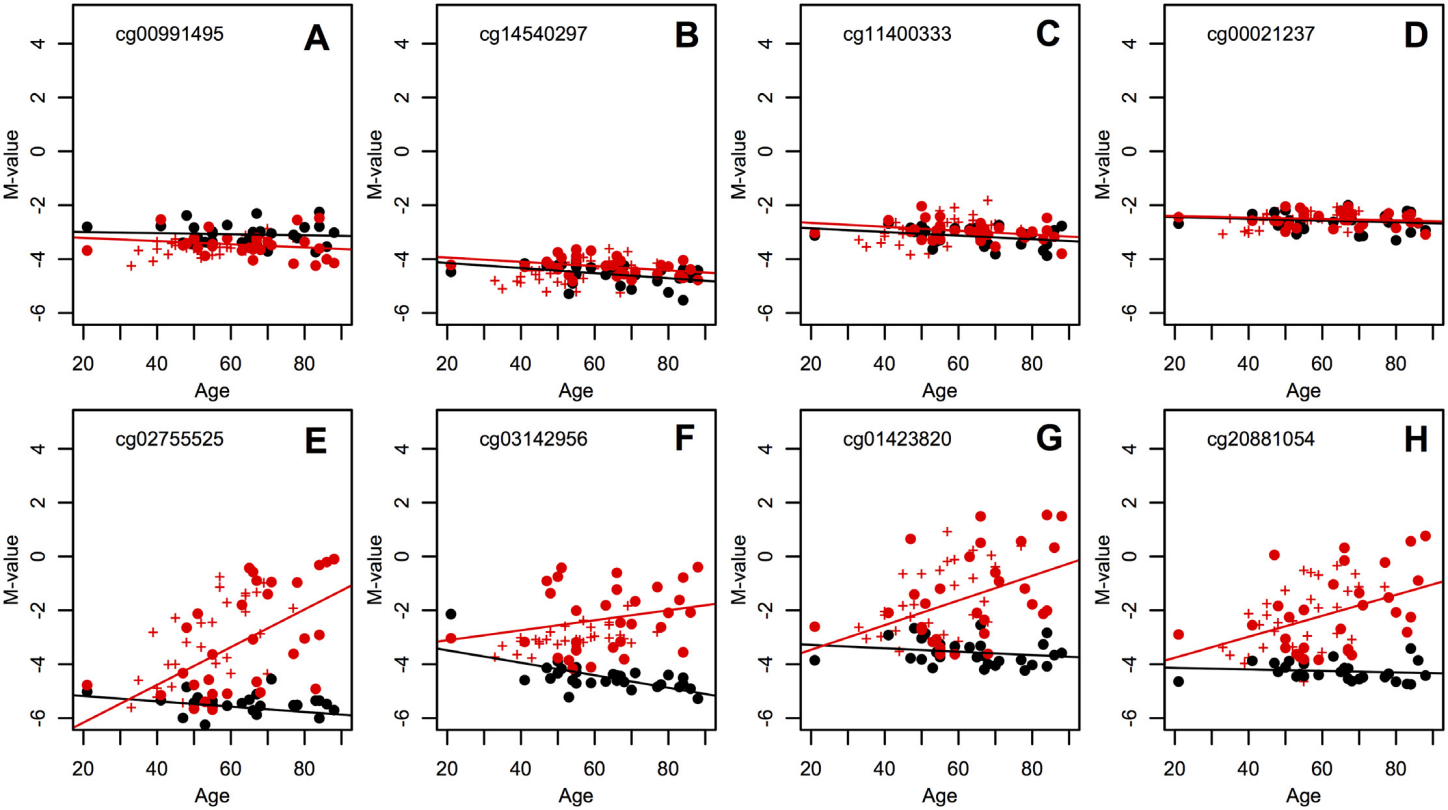
Cross-sectional data and BE clock CpGs. Cross-sectional patient data D2, in which matched squamous (SQ) M-values (black points) and BE M-values (red points) are plotted at corresponding age of biopsy. (A-D) Top row shows 4 of 400 total hypomethylated CpG sites, in which the regression rates of SQ and BE across individuals is not significantly different (p-value = 0.5). In contrast, (E-H) the bottom row shows 4 of the 67 BE clock CpGs with highest p-values for significant individual BE drift from the longitudinal data set D1. The BE clock CpGs are chosen to have significant BE drift differing from SQ drift (p-value = 0.05 with ANCOVA) and large heterogeneity around the population average due to heterogeneous BE onset ages (see Material and Methods). BE data for the longitudinal patients in data set D1 (designated by ‘+’ signs, as in Fig 1) show consistency between the two data sets. Corresponding regression lines for cross-sectional data D2 are also plotted.

Next, we examined which of the candidate CpGs identified in Step 1 show significant differential drift between the matched SQ and BE tissues of data set D2. We used Analysis of Covariance (ANCOVA) regression modeling to test whether the methylomic drift rates (or regression slopes) differed between SQ and BE tissues among the 30 patients in set D2. Specifically, for each marker *j*, we regressed M-values derived from SQ and BE samples onto patient age with histology (SQ or BE) as a categorical variable, i.e., M-value_*j*_ ∼ age ⋅ histology. We divided the candidate CpGs discovered in Step 1 into two subgroups: CpGs that are essentially hypomethylated in SQ tissue with *β*_*SQ*_ < 0.25 (400 CpGs), and those that can be considered hypermethylated in SQ tissue, i.e., *β*_*SQ*_ > 0.75 (274 CpGs) for all SQ samples in D2. See example CpGs from the hypomethylated subgroup in Fig 3. As we will show, this categorization distinguishes positive and negative methylomic drift in BE tissue, respectively for hypo- and hypermethylated CpGs in the reference SQ tissue. This particular choice is less confounded by heterozygous methylation where drift could occur in opposite directions (e.g., when the paternal allele is unmethylated, but the maternal allele is methylated). Using ANCOVA, we found 75/400 CpGs to drift differentially between BE and SQ in the first group (nominal p-values <0.05), while only 14/274 CpGs appeared to drift differentially between the two tissues in the second group. As expected, the majority (67) of the 75 differential, upward drifting CpGs have estimated BE drift rates that are in fact larger than the corresponding SQ drift rates, while only 3 out of 14 differential, downward drifting CpGs have estimated BE drift rates that are lower than the corresponding SQ drift rates. Thus, we will continue our analysis and selection using the larger subset of 75 positively drifting CpGs.

**Fig 4:**
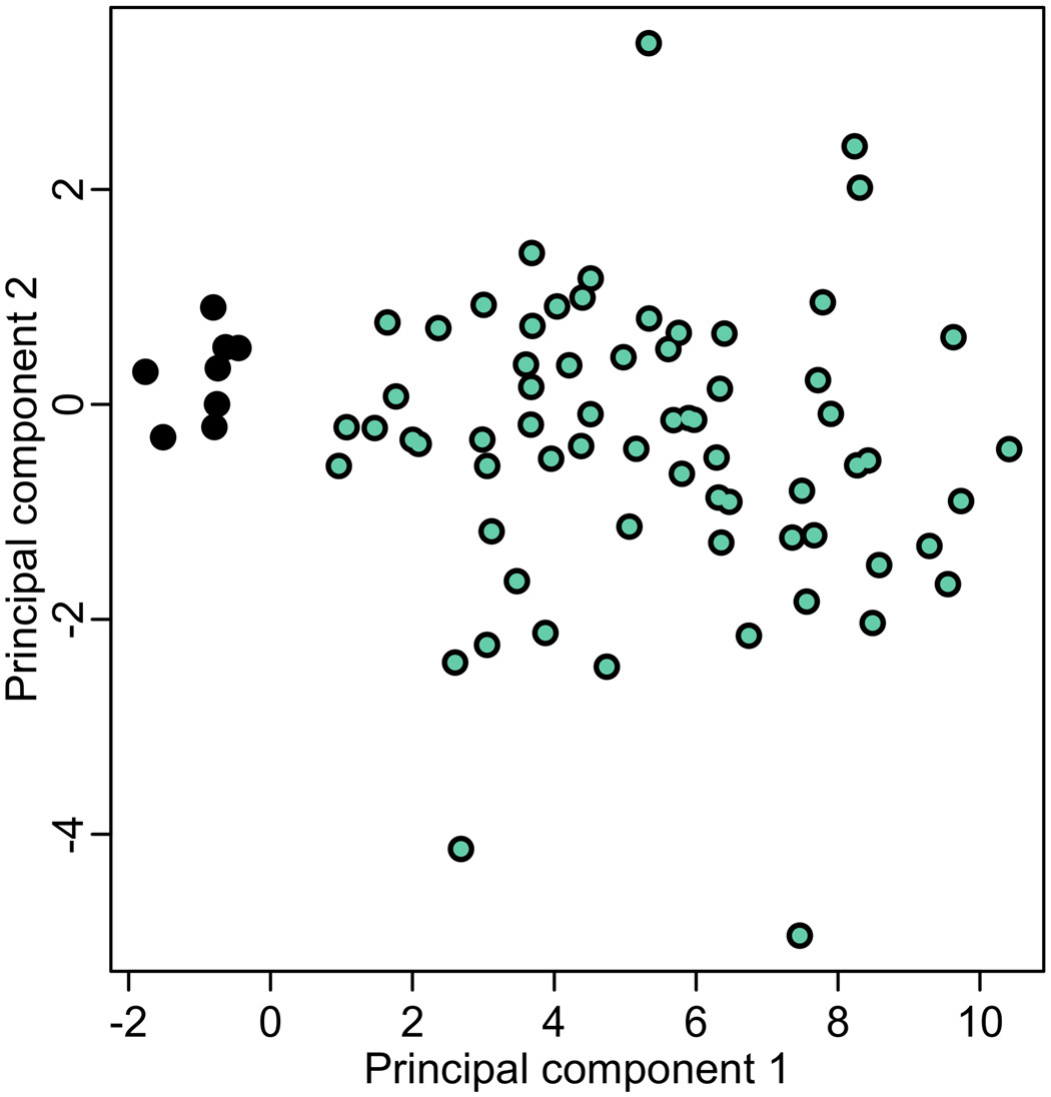
Principal Component Analysis. PCA analysis of regression residuals for 75 differential drift CpGs identified from data set D2. The green points designate the 67 BE clock CpG set. See text for details.

A principal component analysis (PCA) of residuals from the BE methylation age regression (which are hypothesized to reflect BE tissue age differences) for the selected 75 differential, upward drifting CpGs confirms the clustering of these CpGs into one group (67 CpGs) with cross-sectional BE drift rates that are estimated to be higher than those estimated for SQ tissue, which tend to be flat. Only a few outliers (8 CpGs) show the opposite behavior and likely represent false positives from the initial candidate selection in Step 1 (see Fig 4). Thus, we consider the remaining 67 differential drift CpGs as an admissible subset that provides the desired differential methylation information for a quantitative estimation of BE onset times. Additional information on each of the 67 CpGs is provided in S2 Table. In the following we will refer to this specific subset as *BE clock CpGs*. We also find that the qualities of our 67 BE clock CpGs are robust in terms of number of CpGs used for the BE onset estimation (see S2 Fig).

Fig 3E–3H shows data (M-values) from patient data sets D1 and D2 for 4 of the 67 BE clock CpGs. Next, we show how the individual BE onset times can be estimated from the methylomic drift observed in these clock CpGs using a Bayesian model that allows for measurement error and uncertainty in marker-specific BE drift rates.

### Bayesian BE clock model for estimating onset times and drift

Here we show how information about methylomic drift characteristic of BE and differential between BE tissue and normal squamous (SQ) tissue can be combined with individual-level methylation data at a given age to predict when a patient developed BE assuming there is a single time point of origin for BE. Our model (described below) employs Bayesian inference to derive dates of BE onset via initial differential drift away from squamous methylation values, and in this way our method can be considered somewhat analagous to dating divergence times in phylogenies with a relaxed molecular clock [32]. In the following we assume that methylomic drift is essentially linear with age (at the logit scale), although there is also evidence that age-associated variation in methylation levels may be better modeled by a function of logarithmic age for younger individuals [23]. However, this approach has the flexibility to accommodate non-linear drift.

For patient *i*, *i* = 1, …, *N*, the data consist of measurements *y*_*BEi*,*j*_(*t*_*i*_) for BE clock CpG_*j*_ (*j* = 1, …, 67) at observation time (age) *t*_*i*_ = *a*_*i*_. We consider the following linear drift model for the conditional expected methylation values of variable *Y*_*BEi*,*j*_(*t*_*i*_), taken from patient *i* at time *t*_*i*_ for each clock CpG, given the onset of BE occurred at time *s*_*i*_ ≤ *t*_*i*_,

$$E\left[Y_{BEi,j}\left(t_{i}\right)\right]=\alpha_{{}_{SQj}}+b_{{}_{SQj}}s_{i}+b_{i,j}\left(t_{i}-s_{i}\right),$$ (1)

for *j* = 1, …, 67. Thus, given the following parameters—the onset of BE at time *T*_*BE*_ = *s*_*i*_, the rate (*b*_*SQj*_) and intercept (*α*_*SQj*_) of the SQ population regression lines obtained from individuals with matched samples in data set D2, and the patient-specific, CpG-specific BE drift rate *b*_*i*,*j*_—we observe 67 independent measurements for *N* independent individuals. Furthermore, we used the linear regression slopes and intercepts provided by the ANCOVA procedure using the normal squamous sample group in D2 to impute *α*_*SQj*_ and *b*_*SQj*_ in D3 for each BE clock CpG, as implemented in the model shown in Eq (1). For this data set, we did not have matched SQ samples but because the methylation values in normal squamous tissue show little variation for our selection of BE clock CpGs, we assumed that the normal squamous tissues behave similarly for non-familial and familial patients. We show that this approach for imputing SQ M-values for non-matched samples is robust in a sensitivity analysis given in Results. Allowing for patient-specific drift rates for the BE clock CpGs, we explicitly model the inter-individual differences in BE drift rates between ‘slow’ and ‘fast’ aging BE tissues relative to the standard clock, which are measured from means and standard deviations of the serial samples.

Again, the observation from a single patient *i*, for *i* = 1, …, *N*, observed at time *t*_*i*_, is of the form

$$y_{i}=\left\{y_{BEi,j},\;\;j=1,\cdots ,67\right\}.$$ (2)

In the Bayesian BE clock framework defined by Eq (1), the likelihood contribution from a single patient observed at time *t*_*i*_ is given by

$$\begin{array}{c} \prod_{j=1}^{67}f(y_{BEi,j})\\ =\prod_{j=1}^{67}f_{N}(y_{BEi,j};\mu_{BEi,j}=\alpha_{SQj}+b_{SQj}s_{i}+b_{i,j}(t_{i}-s_{i}),\sigma_{BEi}), \end{array}$$ (3)

where *f*_*N*_ is the normal density function. For the Bayesian model we further assume uniform priors *p*_*s*_(*s*_*i*_) for the BE onset times *s*_*i*_ (due to the fact that the distribution of BE onset times in the general population is essentially unknown), conjugate gamma priors *p*_*σ*_(*σ*_*BEi*_) for the standard deviation *σ*_*BEi*_ of methylation measurement values using shape and scale parameters fitted to the distribution of non-drifting CpG measurements, and normal prior distributions *p*_*b*_(*b*_*i*,*j*_) for the drift rates *b*_*i*,*j*_, *j* = 1, …, 67, which were derived from the longitudinal data sets with empirical mean and standard deviation (see S1 Text for full expressions of prior distributions).

In order to ultimately simulate the BE onset times *s*_1_, …, *s*_*N*_ from the corresponding patient-specific posterior distributions, let us define the vector *Ψ*_*i*_ = (*s*_*i*_, *b*_*i*,1_, …, *b*_*i*,67_, *σ*_*BEi*_) for patient *i*. Samples of *Ψ*_*i*_ under its posterior distribution for patient *i* will be obtained using Markov Chain Monte Carlo (MCMC). The posterior distribution of *Ψ*_*i*_ given the observation **y**_*i*_ comprised of patient-specific data of the form in Eq (2), for *i* = 1, …, *N*, is given by

$$\pi (\Psi_{i}|y_{i})\propto \text{likelihood}\;\cdot \;\text{prior}$$ (4)

$$=\prod_{j=1}^{67}f_{{}_{N}}\left(y_{BEi,j};\mu_{{}_{BEi,j}},\sigma_{{}_{BEi}}\right)\cdot p_{{}_{s}}\left(s_{i}\right)\cdot p_{{}_{b}}\left(b_{i,j}\right)\cdot p_{{}_{\sigma }}\left(\sigma_{{}_{BEi}}\right).$$ (5)

To estimate the model parameters of this Bayesian BE clock model we used MCMC with Gibbs sampling [33]. All the full conditionals are known distributions. Specifically, for each individual *i*, *i* = 1, …, *N*, we estimated the posterior means, medians, and other quantiles of the BE onset time *s*_*i*_, patient-specific, CpG-specific drift rates *b*_*i*,*j*_, *j* = 1, …, 67, and patient-specific standard deviation of measurements parameter, *σ*_*BEi*_. All MCMC simulations were run for 100K cycles and allowing 1K cycles for burn-in.

### Validation of methylomic drift

**Fig 5:**
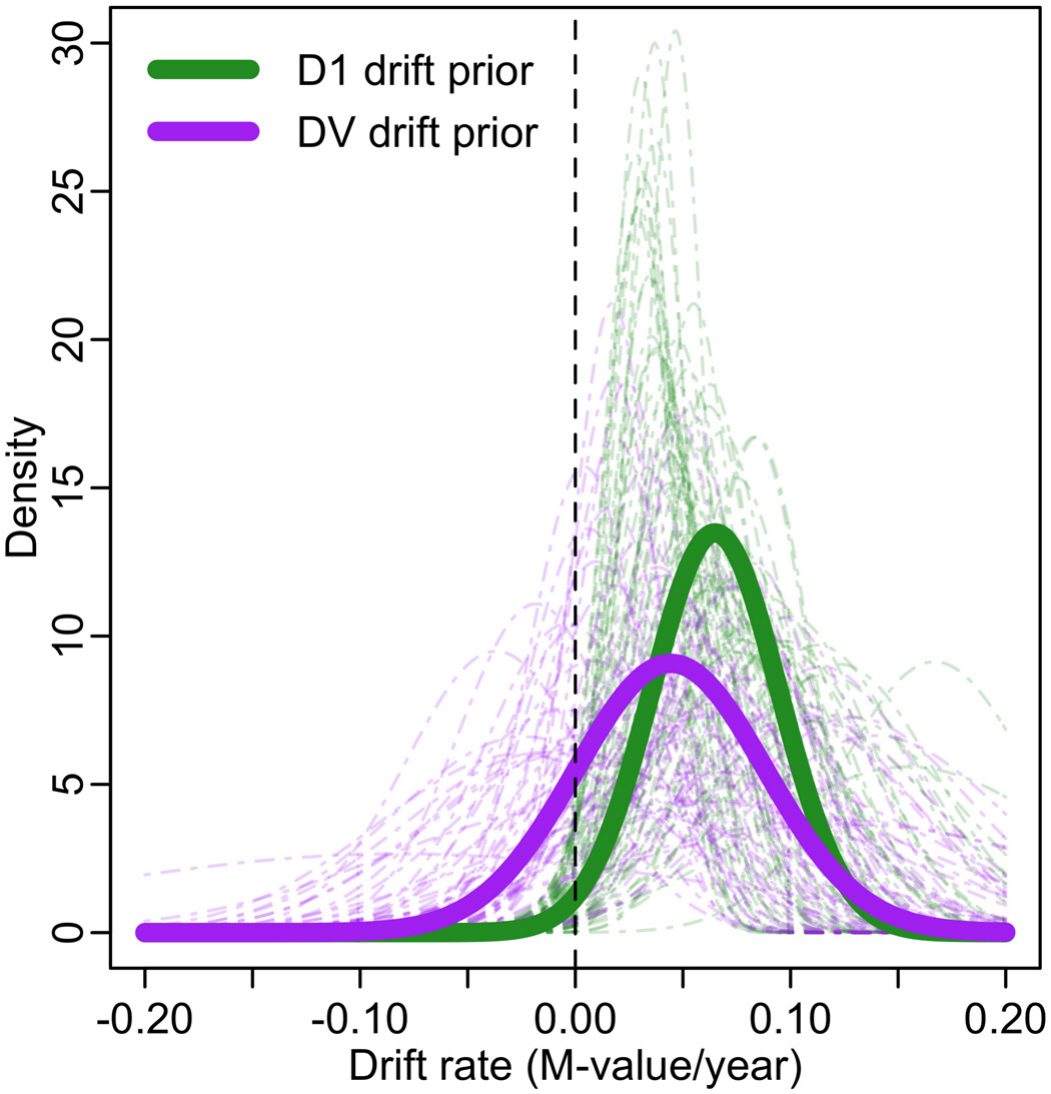
Validation of methylomic drift. Normal distributions derived from regressions using M-values from serial data set D1 across 67 CpGs individually (light dashed green curves) and combined (solid green line). Similar normal distributions derived from regressions using M-values from serial data set DV across these 67 CpGs individually (light dashed purple curves) and combined (solid purple line) are also plotted. Both prior choices from D1 and DV (solid lines) are shifted to the right of zero (vertical dashed black line) depicting validated positive drift in BE tissue for the BE clock CpG set.

The Bayesian BE clock model requires specification of a prior distribution *p*_*b*_(*b*_*i*,*j*_) for the drift rates *b*_*j*_, *j* = 1, …, 67 of the BE clock. In the preselection pipeline described above (Step 1), we obtained mean drift rates (slopes) and standard deviations for each arrayed CpG in the longitudinal study D1. To illustrate the degree of variability and uncertainty in the estimated drift rates we show normal distributions with those means and standard deviations individually (in Fig 5, light dashed green curves) and aggregated as a single normal distribution (solid green curve). To validate the methylomic drift associated with these 67 BE clock CpGs in an independent longitudinal data set (denoted as DV), we used the procedure described in Step 1 to evaluate the drift rates (regression slopes) for each of the 67 CpGs. The results are shown in Fig 5, analogous normal distributions for each of the 67 CpGs in the clock set individually (light dashed purple curves) and in aggregate (solid purple curve) for the validation set DV. S3 Fig shows a scatterplot of mean drift rates between data sets D1 and DV. As expected, overall we observe slightly decreased means and increased variances in the drift rates of the clock CpGs in the validation set DV, a phenomenon commonly referred to as “winner’s curse”, reflecting the typical overestimation of effect sizes in discovery samples (see Fig 5). Ultimately, there was minimal effect of this bias conferred on posterior parameter estimates (see S1 Text).

### Testing significance of BE dwell time differences

In Results, we will apply the Bayesian BE clock model to estimate model parameters for 2 patient data sets independently—cross-sectional (D2) and FBE (D3). To formally assess differences between different patient groups, we use Bayes factors to statistically test if the BE onset ages estimated for one group *s*_*i*_, *i* = 1, .., *N*_*k*_, lead to BE dwell times that are significantly different from those of a second patient set with estimated BE onset ages $s_{i}^{\prime }$, *i* = 1, .., *N*_*l*_, for *k*, *l* ∈ {2, 3}. For two specified data sets *D*_*k*_, *D*_*l*_, we compare the average fraction of life until age at biopsy (*a*_*i*_) during which the patient harbored BE. This quantity is given for two data sets by the following variables,

$$\gamma_{k}=\frac{1}{N_{k}}\sum_{i=1}^{N_{k}}\frac{a_{i}-s_{i}}{a_{i}},\;\;\;\;\;\;\;\;\;\gamma_{l}=\frac{1}{N_{l}}\sum_{i=1}^{N_{l}}\frac{a_{i}^{\prime }-s_{i}^{\prime }}{a_{i}^{\prime }}.$$ (6)

Thus, we are interested in testing hypotheses *H*_0_: *γ*_*k*_ > *γ*_*l*_ versus *H*_1_: *γ*_*k*_ ≤ *γ*_*l*_. For this test, we consider data $y_{∼}=\left\{y_{1},\ldots ,y_{N_{k}},y_{1}^{\prime },\ldots ,y_{N_{l}}^{\prime }\right\}$ comprised of patient-specific observations of the form in Eq (2) and compute the Bayes factor

$$B_{01}=\frac{\mathrm{Pr}[y_{∼}|H_{0}]}{\mathrm{Pr}[y_{∼}|H_{1}]}=\frac{\mathrm{Pr}\left[H_{0}|y_{∼}\right]/\mathrm{Pr}\left[H_{0}\right]}{\mathrm{Pr}\left[H_{1}|y_{∼}\right]/\mathrm{Pr}\left[H_{1}\right]}=\frac{\mathrm{Pr}\left[H_{0}|y_{∼}\right]/\mathrm{Pr}\left[H_{0}\right]}{\left(1-\mathrm{Pr}\left[H_{0}|y_{∼}\right]\right)/\left(1-\mathrm{Pr}\left[H_{0}\right]\right)}$$ (7)

to quantify the evidence in favor of the null hypothesis *H*_0_ and against the alternative *H*_1_ [34]. To compute Pr[*H*_0_|**y**_**∼**_], we apply the ergodic theorem and approximate the posterior probability by the fraction of MCMC samples satisfying *γ*_*k*_ > *γ*_*l*_. The prior Pr[*H*_0_] is computed similarly except we sample onset times *s*_*i*_ for the two groups of patients being compared directly from the uniform prior distributions *s*_*i*_ ∼ Uniform(0, *a*_*i*_).

### Open source code

The methods outlined in this section are implemented by the Bayesian BE clock model. All necessary tools to employ this model via the Gibbs sampler are available in documented R code at https://github.com/yosoykit/BE_Clock_Model.

## Results

### Bayesian BE clock model estimates for BE patients in D2

**Fig 6:**
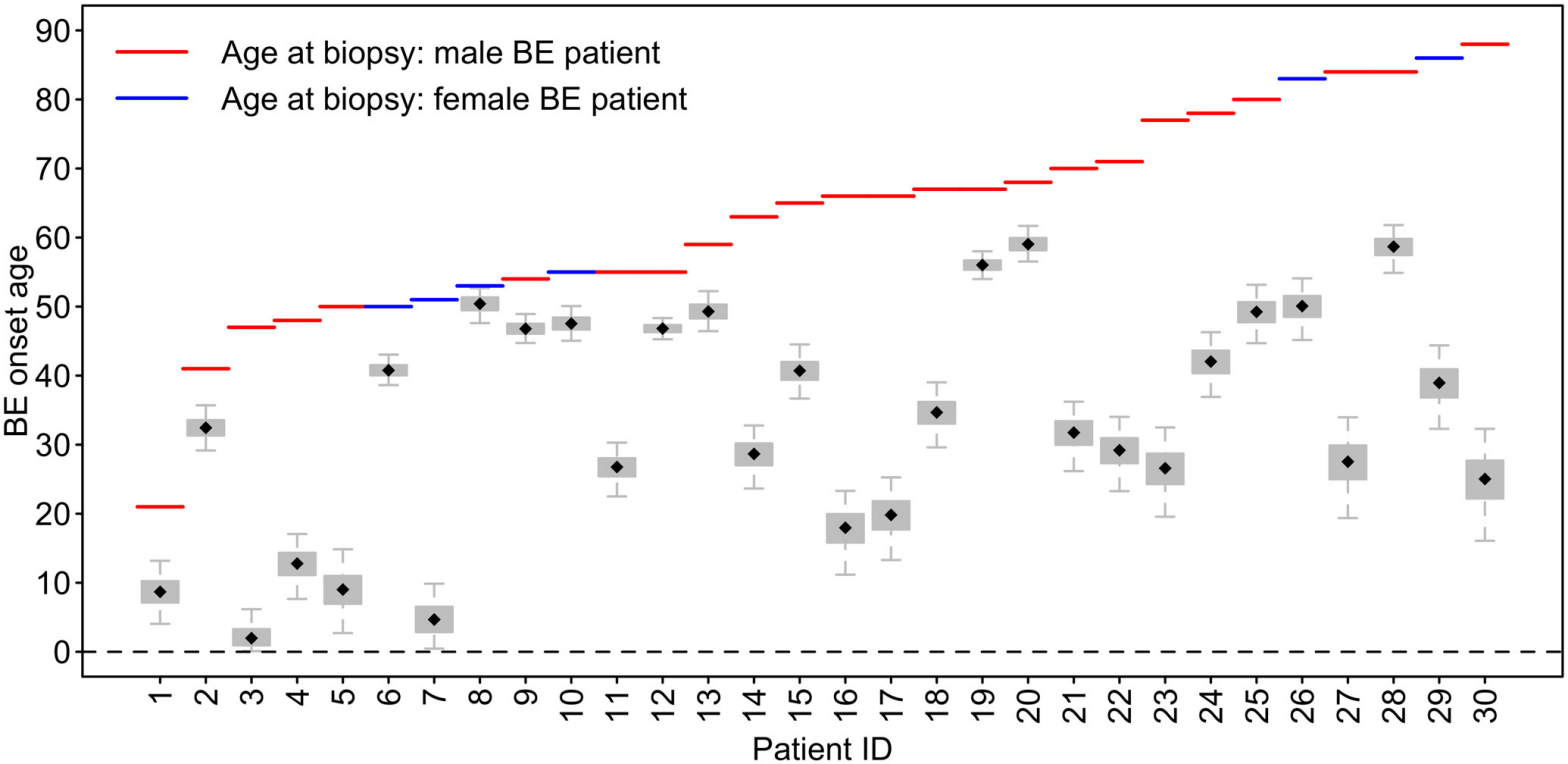
Predicted BE onset times for 30 BE patients in D2. Boxplots depict the MCMC simulated posterior BE onset time distributions for all 30 patients, *s*_*i*_, *i* = 1, …, 30, in data set D2. The MCMC estimates suggest large inter-individual heterogeneity in BE onset times (median onset age = 33.6, range = [2.0, 59.0]), which translates into widely varying EAC risk predictions between patients.

First, we used the Bayesian BE clock model to obtain posterior estimates of parameters for data set D2 (size *N*_2_ = 30 patients) with the BE clock set of 67 CpGs. See Materials and Methods for modeling details and CpG selection. Fig 6 depicts the wide inter-individual variability in the predicted BE onset ages among the 30 patients in D2, with interquartile and 95% credible intervals (CIs) denoted by box and whisker, respectively, for each Markov Chain Monte Carlo (MCMC) parameter estimate of BE onset age *s*_*i*_, *i* = 1, .., *N*_2_. For these 30 patients, median MCMC estimates for BE onset ages ranged from 2.0 to 59.0 years of age, with a median of 33.6 years of age. The model also estimates CpG specific drift rates *b*_*i*,*j*_, *j* = 1, .., 67 for the BE clock set and a measurement standard deviation parameter, *σ*_*BEi*_ for each individual *i* (see Materials and Methods for details).

The BE onset age estimates for the patients in D2 were obtained using prior *p*_*b*_(*b*_*i*,*j*_) derived from data set DV (purple curve in Fig 5). We provide MCMC results when using this prior because 1) the estimates of BE onset times *s*_*i*_, *i* = 1, …, *N*, using the DV prior are very similar to those when using the D1 prior, and 2) the DV prior introduces no bias (i.e., more realistic overall population drift distribution) because it was not used for the BE clock CpG marker set selection.

#### Sensitivity analyses

To investigate the sensitivity of the estimated BE onset ages on the number of CpGs used in the MCMC algorithm we randomly subsampled smaller sets (n = 5 and 20) from the full set of 67 identified BE clock CpGs. We found our estimates of BE onsets to be robust in terms of the number of BE clock CpGs needed to discriminate among patients of similar chronological age who reveal rather distinct (early versus late) BE onset estimates (see the example given in S2 Fig).

Note, Eq (3) assumes independence of the observations given the BE onset time s. This assumption may in fact be violated within CpG islands due to non-local effects in DNA methylation maintenance. To test whether the presence of multiple CpGs on the same CpG-island leads to a bias or significant deflation of the posterior CIs of the BE onset estimates, we completely removed island-level multiplicities by randomly selecting a single CpG per island (including shore and shelf). There are 51 unique islands and one category for CpGs that are not associated with an island that contribute to our clock set. A comparison of the posterior means and CIs of the BE onset times (data set D2) using this construct against randomly chosen control sets of the same size (i.e., 52 CpGs), we find no evidence of bias in the BE onset estimates, nor any significant inflation of the CIs (Welch’s two-sample t-test: p-value >0.9).

Lastly, we tested whether our approach of using the inferred SQ drift from linear regression rather than the patient-specific SQ-matched samples themselves would lead to any loss of information with respect to the estimated BE onset ages. To do this, we used the difference in M-values between BE and SQ, Δ, as the observations in an analogous model (see S1 Text for full analysis) and found that the root-mean-square error in BE onset age estimates was less than one year across D2 patients (see S4 Fig). Thus, our method is robust and suitable for use with BE patient data that does not include SQ-matched tissue, such as data set D3.

### BE onset predictions for familial BE cases in D3

**Fig 7:**
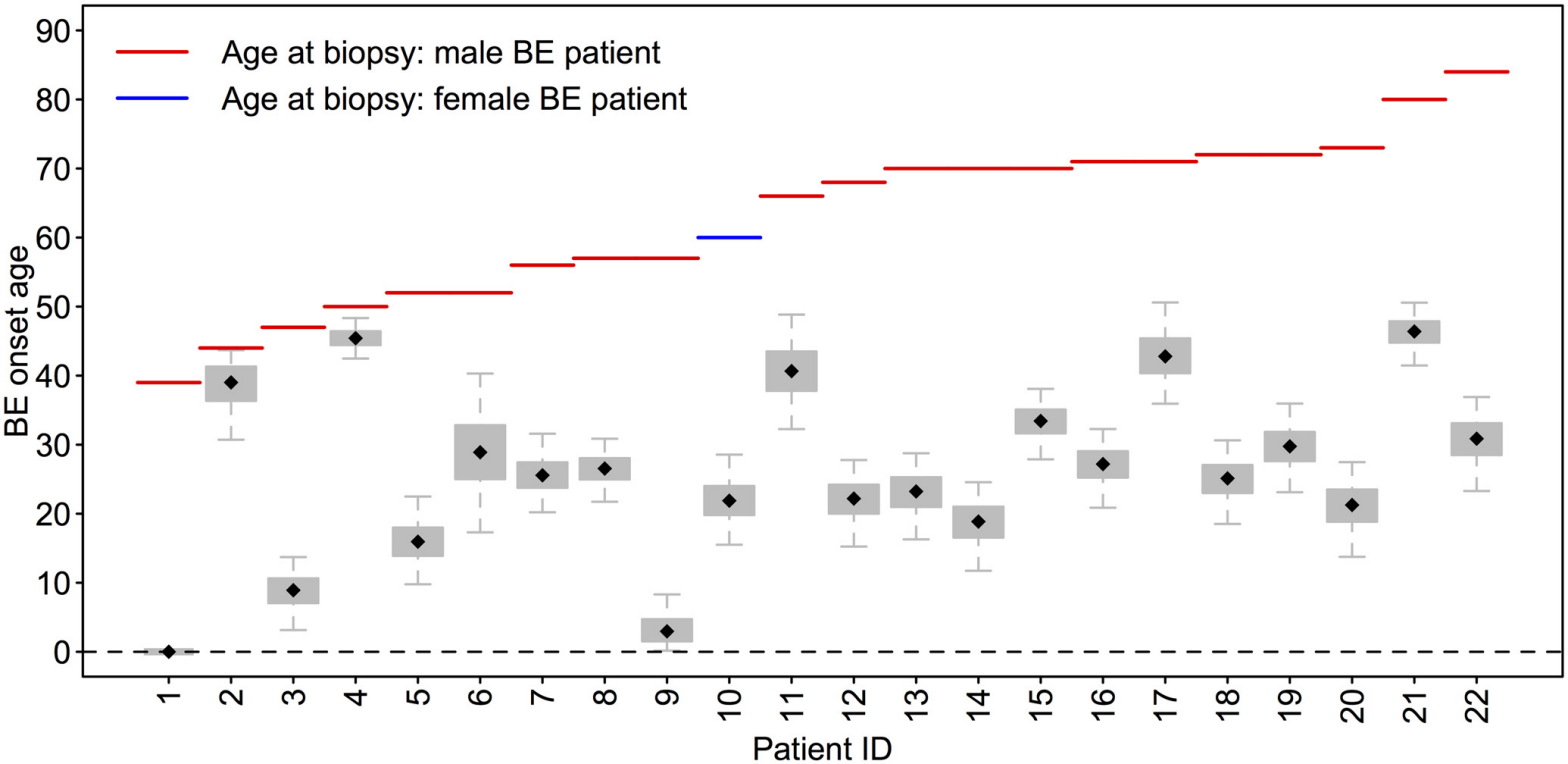
Predicted BE onset times for familial BE patients in D3. Boxplots depict the MCMC simulated posterior BE onset time distributions for all 22 patients, *s*_*i*_, *i* = 1, …, 22, in data set D3. Testing via the Bayes factor suggests that the difference between average BE dwell times for FBE patients versus average BE dwell times for BE patients in data set D2 (see Fig 6) is highly significant.

To quantify the aggregation of BE and EAC in families, Chak et al. performed a study with 411 patients with BE and/or its associated cancers, and found that familial BE (FBE) can be determined in 7.3% of patients, comprising 9.5% of EAC cases [22]. One hypothesis is that FBE patients have a stronger predisposition to develop BE compared to non-familial individuals, possibly due to inherited susceptability gene(s). We estimated the Bayesian BE clock model parameters for the independent data set D3 (size *N*_3_ = 22 patients) with FBE, with age range 39–84 at time of biopsy (mean age = 62.7). Fig 7 depicts the posterior median BE onset ages estimated for the 22 patients in D3, with interquartile and 95% credible intervals denoted by box and whisker, respectively. For these 22 patients, median MCMC estimates for BE onset ranged from 0 to 46.4 years of age, with a median of 26.1 years of age. The youngest FBE patient is shown to have onset at birth due to the incongruence of the standard clock drift rate distribution with his methylation values for the molecular clock set and thus we were unable to obtain positive posterior estimates of his onset age.

**Fig 8:**
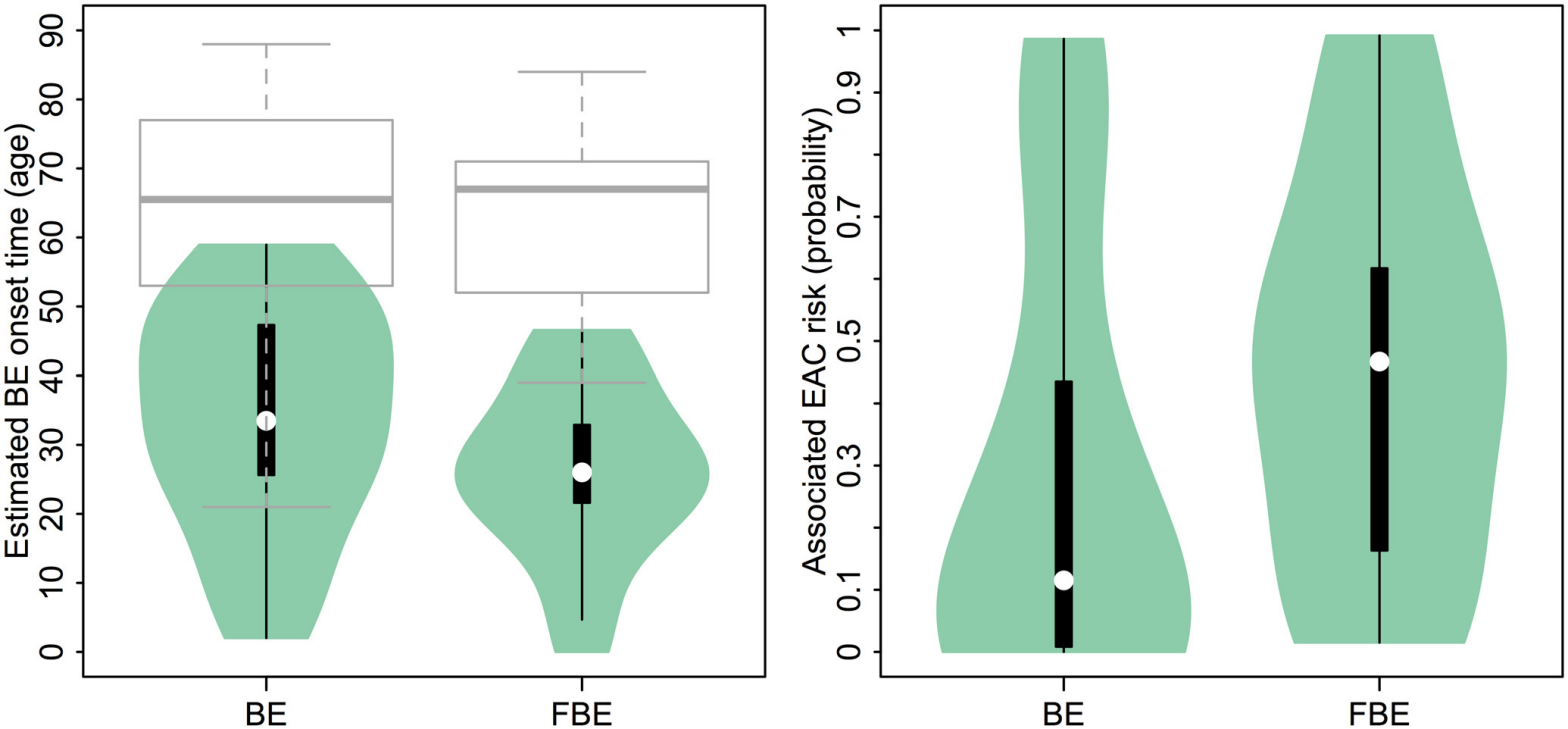
Comparison of Predicted BE onset times and dwell times. (Left panel) Violin plot depicts distributions of median posterior estimates for BE onset times for *N*_2_ = 30 sporadic BE patients in D2 and *N*_3_ = 22 familial BE patients in D3, respectively. The boxplots for similar age-at-diagnosis (i.e., age-at-biopsy) for the two groups is also provided (grey boxplots). (Right panel) Violin plot depicts distributions of EAC risk given median BE onset age estimates for general BE patients in D2 and FBE patients in D3, respectively.

Because a younger age of disease onset is often considered a surrogate marker for a genetic or environmental predisposition, we tested the hypothesis that the FBE patients of data set D3 had been living with their BE for longer than the general BE patients in data set D2, which in our notation translates to *H*_0_: *γ*_3_ > *γ*_2_ (see Materials and Methods for details). The Bayes factor (see Eq (7)) was conservatively estimated to be 100K. This result provides decisive support in favor of the hypothesis that the FBE patients indeed harbored BE (relative to their ages when biopsies were removed for analysis) longer than the general BE population harbored BE (see left panel of Fig 8 for violin plot depicting this result).

### Predicted EAC risks for BE patients

With the BE onset predictions provided in the previous results, we are in a position to associate a patient-specific risk of developing EAC before a certain age. We computed the cumulative risk of developing EAC for each patient before age 88 (age of the oldest patient in our data sets) by using tissue age biomarker data to inform the modeling of the neoplastic progression to EAC. Such an integrated perspective for cancer risk management has recently been suggested by Li and colleagues [35]. To this end, we employ a mathematical model for EAC progression, termed the multistage clonal expansion for EAC (MSCE-EAC) model, that was previously calibrated to EAC incidence in the US by birth cohort, to obtain EAC risk estimates for each patient assuming that all patients share similar risk factors (e.g., unknown dysplasia status at time of biopsy) for EAC progression [8, 16]. Specifically, for each BE patient who has not been diagnosed with EAC by age *a*, given estimated BE onset time *T*_*BE*_ = *s*, we computed the following risk

$$\mathrm{Pr}\left[T_{{}_{EAC}}<88|T_{{}_{BE}}=s,T_{{}_{EAC}}>a\right]=\frac{S_{{}_{MSCE}}\left(a-s\right)-S_{{}_{MSCE}}\left(88-s\right)}{S_{{}_{MSCE}}\left(a-s\right)},$$ (8)

where *S*_*MSCE*_ is the EAC survival probability for the multistage clonal expansion (MSCE) model after BE initiation (see S1 Text for a derivation and S1 Fig for a model schematic) [8, 16, 36]. Alternatively, we may use summary (constant) risk estimates of progressing from non-dysplastic BE to EAC using published annual risk estimates across individuals of different age and different BE onsets. Note, however, for general *s* < *a* our mathematical EAC model implies the following inequality,

$$\mathrm{Pr}\left[T_{{}_{EAC}}<88|T_{{}_{BE}}=s,T_{{}_{EAC}}>a\right]\neq \mathrm{Pr}\left[T_{{}_{EAC}}<88|T_{{}_{BE}}<a<T_{{}_{EAC}}\right],$$ (9)

which demonstrates that a patient’s BE onset adds information to refine blanket risk stratifications that do not consider this information.

As a demonstration, we used this model to compute the patient-specific risk of developing EAC by age 88 assuming a standardized 1950 birth cohort, allowing for gender-specific model parameters, by inputting the BE onset age estimate *s* for each patient into Eq (8). See S1 Table for the MCMC BE onset median estimates (with 95% credible intervals) of the 2 BE data set groups. Fig 8 shows the distributions of median MCMC estimated BE onsets for the 2 patient data sets (green violin plots) and their age-at-biopsy distributions (grey boxplots), alongside the corresponding EAC risk estimates for these onset ages. Of the two patient groups, the FBE patients in data set D3 have a significantly higher predicted median EAC risk estimate of 0.47 compared to the sporadic BE population with a median risk of 0.11. Because EAC risk is predicted by our model to increase monotonically with BE dwell time for patients of the same age, the correlation between estimated BE onset age and predicted EAC risk by age 88 is very high across patients (corr = .92 for data set D2, corr = .97 for data set D3, see S5 Fig).

## Discussion

A fundamental problem in predicting the risk of esophageal adenocarcinoma (EAC) in patients with BE continues to be the difficulty in assessing the neoplastic potential of BE, which is considered the premalignant field in which EAC arises. Several lines of evidence and theoretical considerations support the notion that both BE segment length and the duration of how long BE has been present in a patient (i.e., BE dwell time) are important determinants of EAC risk in addition to environmental and genetic risk factors [16, 37, 38]. While endoscopic surveillance with systematic biopsy sampling is the standard clinical care to screen BE patients for dysplasia and early cancer, most BE patients never develop esophageal cancer in their lifetimes. Priority has therefore been given to novel approaches to identify the molecular signatures of EAC progression and biomarkers in an attempt to more precisely define EAC risk at an individual level. However, because chronological age is recognized as one of the strongest predictors of cancer risk, renewed attention has been given to exploring the roles of biological tissue-age and cellular senescence in the progression to cancer [39].

Unfortunately, a clinical determination of when a patient first developed BE is presently not possible because BE is mainly asymptomatic (over 90% of EAC cases do not present with a prior history of BE [40]). For this reason we made an attempt to validate our BE onset predictions indirectly through two lines of evidence. First, we validated the longitudinal drift rates with an independent data set (DV). Although the drift rates for the BE clock set were generally lower in the validation set DV compared with the rates seen in set D1 (which we attribute to selection bias in D1), we found very similar estimates of the BE onsets using either drift-rate prior distribution in our Bayesian model. Secondly, we considered previous efforts to identify tissue-based indicators that accurately reflect the biological age of a tissue using regularized regression techniques by directly regressing age on the levels of methylation at a large number of CpGs to identify subsets of CpGs that are predictors of chronological age [13, 14]. Although we cannot use these techniques in this context because the BE onset times are unknown, we find that our predictions are at least broadly consistent with the straightforward application of these clock models to estimate absolute tissue-age differences between BE and SQ tissue. Specifically, using the published elastic net coefficients by Horvath [14] and by Hannum et al. [13] we computed the predicted biological age of the BE tissue and subtracted the predicted biological age of the normal squamous (SQ) esophageal tissue to arrive at crude estimates of the BE dwell time for the 30 patients in D2 (the cross-sectional cohort of patients). By subtracting these estimates from the chronological ages of the patients we obtained corresponding BE onset times that correlated well with our predictions (r = 0.77 for the Horvath 110 clock-CpG model, r = 0.84 for the 89 clock-CpG model by Hannum et al.).

Finally, we tested our clock model using methylation array data from 22 familial BE patients (set D3). Patients from both groups D2 and D3 have similar age distribution (see Fig 8 and S1 Table). However, compared to the onset ages estimated for the patients in data set D2, the familial group show increased BE dwell times; Bayes factor testing for the FBE study suggests that the inferred BE onset times, although heterogeneous (Fig 8), tend to occur significantly earlier in life for FBE patients compared to nonfamilial BE cases implying a possible heritable predisposition to develop BE metaplasia. Given that the predictions of BE onsets among FBE cases are significantly earlier than the predictions for the sporadic cases, it is perhaps surprising that the age distribution for the familial cases is not dissimilar to the age distribution for the sporadic cases (see grey boxplots in Fig 8). One possible explanation is that, next to symptomatic reflux, heartburn and other common risk factors, family history may not have been an indicator for referral to endoscopy as familiarity of this disease was only discovered in the past couple decades [22]. Therefore, if reflux frequency and other indicators for referral are similar for familial and non-familial patients, we expect the mean ages of BE diagnosis to be similar between the two groups. Specifically, we found the median estimates of BE onset age for the FBE patients to be 7.4 years earlier on average than the sporadic BE cases in study D2. This result is consistent with the result of a large study by Chak et al. that concluded that multiplex FBE families (multiplex being defined as having at least 2 confirmed FBE cases among family members) develop EAC at an earlier age compared with nonfamilial EAC cases [38]. Similar to the conclusions drawn by these authors, our result suggests that FBE patients may need earlier and possibly more frequent endoscopic screening for neoplastic lesions in BE tissue before EAC develops.

Given the theoretical implications of our proposed model of BE initiation and progression to EAC, we propose that once a patient’s BE onset has been estimated from his/her methylomic drift profile, his/her risk of developing EAC can be estimated more precisely. We have used a previously validated multistage clonal expansion model for EAC incidence which explicitly considers the uncertainty of the timing of BE onset in the general population and describes, conditional on when BE develops, the stochastic process of neoplastic progression from metaplastic to dysplastic tissue to cancer [8, 16]. These theoretical predictions show a strong dependence of EAC risk on the BE dwell time (see Fig 8). Importantly, we found that the lifetime risks for the individuals in study D2 vary widely, with an interquartile range of 0.01 to 0.44. It is important to recognize that these EAC risk predictions do not consider the effects of interventions and therefore may be overestimates. Although this predicted variability in risk stands unconfirmed, our median risk prediction of 0.11 for the D2 patients (see Fig 8) is consistent with empirical estimates of the EAC lifetime risk in BE patients found in the range 0.07–0.13 [41]. Therefore, the finding that the lifetime risks for the individuals in study D2 vary widely with an interquartile range of 0.01 to 0.44 translates into relative EAC risks (for the 4th quartile relative to 1st quartile) of > 40, assuming an otherwise homogenous population. For comparison, we found positive correlations between our D2 EAC risk predictions based on BE onset and D2 EAC risk estimates using previously reported risk factors based on gender (corr = 0.57, p = .001), histopathological grade (corr = 0.53, p = .003), and chronological age (corr = 0.49, p = .006) [6]. However, each of those risk factor estimates led to much lower relative EAC risks of <3. This suggests that BE onset, as determined by methylomic drift, can be considered a potential biomarker for EAC risk, although further validation via properly powered prospective studies or case-control studies in BE patients are needed to confirm this. Such studies may provide the requisite data to further test how well BE tissue-age performs in identifying individuals that likely progress to HGD or EAC in their lifetime so that endoscopic surveillance and available interventions can be utilized more effectively.

## Supporting Information

S1 Text Mathematical details of clock model and predicted patient-specific EAC risk. Explicit distributions used in the Markov Chain Monte Carlo (MCMC) inference and an analysis for robustness of imputing normal squamous M-values is provided. For the multistage clonal expansion for EAC (MSCE-EAC) model, we derive the equation for EAC risk given a patient’s BE onset age from the backward Kolmogorov equations corresponding to the multi-type branching process [16].
(PDF)

S1 Fig The multistage clonal expansion for EAC (MSCE-EAC) model. Normal squamous epithelium may transform to BE with an exponentially distributed onset time with rate *ν*(*t*), followed by a ‘two-hit’ tumor initiation process with Poisson initiation rates *μ*_0_, *μ*_1_, which leads to the stochastic appearance of premalignant progenitor cells in the tissue. Premalignant cells undergo a first clonal expansion described by a birth-death-migration process with cell division rate *α*_*P*_, cell death-or-differentiation rate *β*_*P*_, and malignant transformation rate *μ*_2_. Malignant cells, in turn, undergo a second clonal expansion by a birth-death-detection process with cell division and death rates *α*_*M*_ and *β*_*M*_, respectively, allowing for stochastic growth and possibly extinction of the malignant tumor. Clinical detection occurs through a size-based detection process with parameter *ρ*. TSG, tumor suppressor gene [16].
(TIFF)

S2 Fig Robustness of the number of CpGs in the BE clock set. Comparison of the posterior distributions of BE onsets for two 84 year old BE patients (pt. 21 and pt. 28) in study D2 using the identified set of 67 BE clock CpGs (thin solid line). To test the relative robustness of the estimated mean BE onsets, we also generated random subsamples (without replacement) of size 5 and 20 from the 67 clock CpGs. Shown are the distributions of the median BE onset estimates using MCMC (5K cycles) for n = 5 CpGs (thick solid lines) and n = 20 CpGs (dashed line).
(TIF)

S3 Fig Scatterplot of mean drift rates between data sets D1 and DV. Between the entire sets D1 and DV, we see relatively low correlation for mean marker-specific drift rates calculated via linear regression (see Methods). However, this plot does suggest that there are outliers (negative *b*_*j*_ rates in DV colored in red) that hide an interesting correlation. Rather than homogenous drift, the correlation between longitudinal drift rates in D1 and DV (with outliers removed, corr = 0.45, p-value < 0.05) suggests the presence of heterogeneity in marker-specific drift rates. Ultimately, there was minimal effect conferred on posterior parameter estimates due to “winner’s curse” bias inherent in the D1 drift rates calculated during BE clock marker selection versus validation DV drift rates when used as two candidate priors in the MCMC (see S1 Text).
(TIFF)

S4 Fig Robustness of BE onset estimation using imputed squamous drift. For the unmasked (grey boxplots) and masked (purple boxplots) implementations of inferring BE onset ages, we found that using an imputation of the intercept and drift rates of SQ tissue values across the D2 patients rather than exact matched SQ values is a robust approach (see S1 Text for details). Specifically, the correlation of median estimates between the two methods was .98, and the root-mean-square error between onset ages was 0.08 years.
(TIFF)

S5 Fig Predicted EAC risk by age 88 given BE onset age estimates. Across patients in data sets D2 (blue points) and D3 (black points), there is high correlation between the median MCMC posterior estimates for BE onset age and the corresponding EAC risk before age 88 as predicted by the multistage clonal expansion model (S1 Fig) that utilizes BE onset as an input. The stochastic model predicts that risk increases exponentially with earlier BE onset ages for patients of similar age. Square points designate males, triangle points designate females.
(TIFF)

S1 Table BE patient information for 5 independent data sets. Patient-specific information (72 total) for 10 serially sampled BE patients (D1), 10 serially sampled patients in an independent validation cohort (DV), 30 cross-sectional BE patients (D2), and 22 familial BE (FBE) patients (D3). Age at biopsy, sex, and whether a matched normal squamous (SQ) tissue sample was obtained at time of biopsy is recorded for all patients. Also, median MCMC estimates (with 95% credible intervals) for BE onset times are provided for cross-sectional patients.
(XLSX)

S2 Table (Epi)genetic information for BE clock CpG set. For 67 total CpGs in BE clock set, columns of this table (in order) correspond to CpG name, gene location of CpG (IGR: intergenic region), chromosome location of CpG, CpG island type location (“‘OpenSea” indicates that the CpG does not lie in a CpG island), CpG island name, whether the CpG is on a promoter region, the CpG-specific population rate from linear regression over 30 D2 samples, and the drift rates for each CpG derived from the D1 and DV patients, respectively. The DV prior drift rates were used as prior information in the BE clock model.
(XLSX)
