# Correction: A Molecular Clock Infers Heterogeneous Tissue Age Among Patients with Barrett's Esophagus

**DOI:** 10.1371/journal.pcbi.1005439

**Published:** 2017-03-17

**Authors:** The PLOS Computational Biology Staff

The Funding section is incomplete. The full statement should read: “This research was supported by the National Institutes of Health (www.nih.gov) and the National Cancer Institute (www.cancer.gov) under grants U01CA182940 (BG-U01) (to EGL, CJW, WDH, WMG, and KC), 5P30CA015704 (to WMG and CJW), 5U01CA152756 (to WMG and CJW), 5U54CA163060 (to AC), NIH1P50CA150964-01A1 (to JEW), The DeGregorio Family Foundation (www.degregorio.org) (to WMG and EGL) and the Price Family Foundation (to WMG and EGL). The funders had no role in study design, data collection and analysis, decision to publish, or preparation of the manuscript.”
