# Supplementary material for: A Molecular Clock Infers Heterogeneous Tissue Age Among Patients with Barrett’s Esophagus
Source: PLoS Comput Biol. 2016 May 11;12(5):e1004919. doi: 10.1371/journal.pcbi.1004919 (PMC4864310; doi:10.1371/journal.pcbi.1004919)
Supplement: S1 Text — Explicit distributions used in the Markov Chain Monte Carlo (MCMC) inference and an analysis for robustness of imputing normal squamous M-values is provided. For the multistage clonal expansion for EAC (MSCE-EAC) model, we derive the equation for EAC risk given a patient’s BE onset age from the backward Kolmogorov equations corresponding to the multi-type branching process [16]. (PDF) [file pcbi.1004919.s001.pdf]

Supporting Information (SI) S1 Text:
A Molecular Clock Infers Heterogeneous Tissue Age Among Patients with Barrett's Esophagus

Mathematical Methods

Distributions in Markov Chain Monte Carlo

As described in Materials and Methods in the Main Text, the methylation data for the 67 BE clock CpG set from a single patient i, for $i = 1, \ldots, N$, observed at time t_i, is of the form

$$\mathbf{y}_i = \{y_{BEi,j}, \quad j = 1, \ldots, 67\}. \tag{1}$$

Here we define the following variables: the onset of BE at time $T_{BE} = s_i$, the slope (b_{SQj}) and intercept (α_{SQj}) of the SQ population regression lines (obtained from individuals with matched samples in data set D2 and thus fixed), the patient-specific, CpG-specific BE drift rates $b_{i,j}$, and the standard deviation σ_{BEi} around data measurement values. For each data set of size N patients, we observe 67 independent measurements for each independent individual. In the Bayesian BE clock model framework, the likelihood contribution from a single patient observed at time t_i is given by

$$\prod_{j=1}^{67} f(y_{BEi,j})$$
$$= \prod_{j=1}^{67} f_N(y_{BEi,j}; \mu_{BEi,j} = \alpha_{SQj} + b_{SQj} s_i + b_{i,j}(t_i - s_i), \sigma_{BEi}), \tag{2}$$

where f_N is the normal density function. For those Bayesian model parameters to be inferred, we assume prior distributions $p_s(s_i)$, $p_b(b_{i,j})$, $p_\sigma(\sigma_{BEi})$ to be used in Markov Chain Monte Carlo (MCMC). First, we assume uniform priors $p_s(s_i)$ for the BE onset times s_i (due to the fact that the distribution of BE onset times in the general population is essentially unknown),

$$p_s(s_i) = \frac{1}{t_i}, \tag{3}$$

where individual i was age t_i at time of biopsy and thus developed BE at some previous age s_i.

Second, we assume normal prior distributions $p_b(b_{i,j})$ for the drift rates $b_{i,j}, j = 1, \ldots, 67$,

$$p_b(b_{i,j}) = \frac{1}{\sigma_b\sqrt{2\pi}} \exp\left(-\frac{(b_{i,j} - \mu_b)^2}{2\sigma_b^2}\right), \tag{4}$$

where the empirical mean μ_b and standard deviation σ_b were derived from the longitudinal data sets D1 and DV. For discovery set D1 used during CpG marker selection, we computed from regression slopes that the drift rates for the 67 CpG set have mean rate $\mu_b = 0.0650$ with standard deviation $\sigma_b = 0.0296$. For validation set DV that was not used during selection, the drift rates computed for the same 67 CpG set have mean rate $\mu_b = 0.0444$ with standard deviation $\sigma_b = 0.0439$. As expected, we see selection bias manifested by a slightly increased mean slope and decreased variance in data set D1 because that data was used for marker selection of

significant CpG drift across individuals via regression (see Fig 5 in Main Text). The use of either prior derived from the two drift rate distributions had little effect on our posterior estimates for the parameters, of most interest the BE onset age s_i (s_i medians differed by only 3.5 years on average). Thus, we provide model results throughout the Results section of the Main Text using the unbiased DV set empirical values to construct prior $p_b(b_{i,j})$ for the drift rates.

Third, we assume a conjugate gamma prior $p_\sigma(\sigma_{BEi})$ for the standard deviation σ_{BEi} of methylation measurement values,

$$p_\sigma(\sigma_{BEi}) = \frac{\gamma_2^{\gamma_1}}{\Gamma(\gamma_1)} \exp(-\gamma_2 \sigma_{BEi}), \tag{5}$$

letting shape $\gamma_1 = 0.608$ and rate $\gamma_2 = 0.117$, values that were fitted to the distribution of 'flat', or non-drifting, CpG measurements in the cross-sectional data set D2 (see 4 examples of such CpGs in Fig 2A-D in Main Text).

Finally, in order to simulate patient-specific posterior distributions via MCMC, let us define the vector $\Psi_i = (s_i, b_{i,1}, \ldots, b_{i,67}, \sigma_{BEi})$ for patient i. Samples of Ψ_i under its posterior distribution for patient i will be obtained using an MCMC algorithm publicly made available online (`https://github.com/yosoykit/BE_Clock_Model`). Note that the posterior distribution of Ψ_i given the observation $\mathbf{y}_i$ comprised of patient-specific data of the form in Eq. (1), for $i = 1, \ldots, N$, is given by

$$\pi(\Psi_i|\mathbf{y}_i) \propto \text{likelihood} \cdot \text{prior} \tag{6}$$

$$= \prod_{j=1}^{67} f_N(y_{BEi,j}; \mu_{BEi,j}, \sigma_{BEi}) \cdot p_s(s_i) \cdot p_b(b_{i,j}) \cdot p_\sigma(\sigma_{BEi}). \tag{7}$$

Thus we simulate posterior distribution samples for model parameters of this Bayesian BE clock model using MCMC. With the expressions above, the full conditionals for our Gibbs sampler can be routinely worked out [1].

Robustness of Estimates using Imputed SQ Drift

To analyze the robustness of imputing SQ trends for BE onset age estimation, we consider a slightly modified model to that which is currently given in Eqns. (1-5) in the Main Text. Rather than considering the random variable of interest to be the M-value measurement $Y_{BEi,j}(t_i)$ for each BE patient, the reformulated model considers the *difference*, $\Delta_{i,j}$, in M-values between BE and SQ samples in patient i, CpG j, given by the following,

$$E[\Delta_{i,j}] = \mu_{\Delta_{i,j}} = (b_{i,j} - b_{SQj})(t_i - s_i). \tag{8}$$

Thus, the observation from a single D2 patient i, for $i = 1, \ldots, N$, observed at time t_i, in the unmasked version is of the form

$$\mathbf{y}_i = \{y_{BEi,j} - y_{SQi,j}, \quad j = 1, \ldots, 67\}. \tag{9}$$

In contrast, the method to infer onset ages we use in the Main Text does not explicitly use the matched SQ sample in an individual's MCMC. Specifically, we currently 'mask' the patient-specific SQ M-values by using fixed intercept α_{SQj} and slope b_{SQj} for each CpG j attained from

linear regression across all D2 patients. We then apply this approach to other data sets such as D3 who do not have SQ-matched samples. Analogously written in the difference (Δ) model formulation, this masked version would use observations of the form

$$\mathbf{y}_i = \{y_{BEi,j} - (\alpha_{SQj} + b_{SQj} t_i), \quad j = 1, \ldots, 67\}. \tag{10}$$

Thus, the analog to Eq. (5) in the Main Text would be the following equation for the posterior distribution of parameter vector Ψ_i given the observation $\mathbf{y}_i$ for patient i,

$$\prod_{j=1}^{67} f_N(\mathbf{y_{i,j}}; \mu_{\Delta_{i,j}}, \sigma_{\Delta_i}) \cdot p_s(s_i) \cdot p_b(b_{i,j} - b_{SQj}) \cdot p_\sigma(\sigma_{\Delta_i}), \tag{11}$$

where the normal prior p_b is adjusted to estimate parameter $b_{i,j} - b_{SQj}$ with appropriate mean and standard deviation, and gamma prior p_σ now applies to standard deviation of the sum of two normal random variables, $Y_{BEi,j}, Y_{SQi,j}$, which is $\sigma_{\Delta_i} = \sqrt{2}\sigma_{BEi}$.

We ran the Bayesian BE clock model for all patients in D2 using the unmasked (Eq. (9)) and masked (Eq. (10)) versions of the Δ model above to determine if the lack of paired SQ matched data leads to any information loss in individual BE onset age estimation among the D2 patients. We found that using an imputation of the intercept and drift rates rather than exact matched SQ values is a robust approach to estimating BE onset ages (see S5 Fig for comparison). Specifically, the correlation of median estimates between the two methods was .98, and the root-mean-square error between onset ages was 0.08 years. Thus, there is minimal information loss resulting from a lack of matched SQ tissue samples for the D2 patients. This is an advantageous aspect of our approach for BE dwell time estimation, particularly for future validation opportunities that may be limited to strictly BE tissue samples as was the case for data set D3.

Lifetime EAC Risk Derivation

In the Main Text, we compute the risk of developing EAC by age 88 in a cancer-free individual at time of biopsy/diagnosis a, given estimated BE onset time, with the multistage clonal expansion for EAC (MSCE-EAC) model (S1 Fig) that was previously calibrated to Surveillance, Epidemiology, and End Results (SEER) incidence data [2–4]. This risk is defined as the probability of developing EAC at random time T_{EAC} by age 88 conditional on the onset age of BE, T_{BE}. For each BE patient who has not been diagnosed with EAC at age at biopsy a, with BE onset time estimated to be age s from his/her methylation profile, we computed the following risk

$$\Pr[T_{EAC} < 88 | T_{BE} = s, T_{EAC} > a] = \frac{S_{MSCE}(a - s) - S_{MSCE}(88 - s)}{S_{MSCE}(a - s)}, \tag{12}$$

where S_{MSCE} is the EAC survival probability for the MSCE-EAC model given BE onset (thus simply an MSCE model). This function has been derived previously (see S1 Text of [4]) but for completeness we will provide a derivation here as well. We first introduce the notation for the

following random variables of the multi-type branching process

$$\begin{aligned} BE(t) &= \text{Bernoulli random variable for BE conversion by time } t \\ X(t) &= \text{number of BE stem cells in a tissue at time } t \\ P^{*}(t) &= \text{number of pre-initiated cells at time } t \\ P(t) &= \text{number of premalignant (initiated) cells at time } t \\ M(t) &= \text{number of malignant (preclinical) cells (prior to detection) at time } t \\ C(t) &= \text{number of cancer cells (after detection) at time } t \\ D(t) &= \text{Bernoulli random variable for clinical detection by time } t \end{aligned}$$

Let us consider the probability generating function (pgf) Ψ for the entire process starting at $\tau = 0$, ie. when an individual is born

$$\Psi(y_{BE},y_1,y_2,y_3,z;t)=\sum_{i,j,k,l,n} y_{BE}^{i} y_1^{j} y_2^{k} y_3^{l} z^{n} P(i,j,k,l,n;t),$$

$$P(i,j,k,l,n;t)=\Pr[BE(t)=i,P^{*}(t)=j,P(t)=k,M(t)=l,D(t)=n|BE(0)=0,P^{*}(0)=0,P(0)=0,M(0)=0,D(0)=0]$$

where, explicitly, $i, n = \{0, 1\}$ and $BE(t), D(t)$ are the following indicator functions corresponding to BE conversion and EAC clinical detection, respectively

$$BE(t) = \begin{cases} 0 & \text{if BE has not developed by time } t \\ 1 & \text{if BE conversion has taken place by time } t \end{cases}$$

$$D(t) = \begin{cases} 0 & \text{if no cancer detected clinically by time } t \\ 1 & \text{if a malignant cell is detected by time } t. \text{ ie } C(\tau) > 0 \text{ for some } \tau \leq t \end{cases}$$

The Chapman-Kolmogorov equations governing the transition probabilities for this multistage process include contributions from the initial Armitage-Doll type transition to BE, the two Poisson transitions to initiation, and the two birth-death-migration processes, all of which have been derived previously [5–7]. We begin with a method for solving for these generating functions using the Kolmogorov backward equations.

Backward Kolmogorov equations and the MSCE hazard, S_{MSCE}

Beginning with an active BE segment (BE), a single pre-initiated (P^{*}), premalignant (P), or malignant (M) cell at time τ only, we define the following generating functions Φ_{BE},$\Phi_{P^{*}}$, Φ_{P}, or Φ_{M}, respectively,

$$\begin{aligned} \Phi_{M}(y_3,z;\tau,t) &= E[y_3^{M(t)} z^{D(t)} | M(\tau)=1, D(\tau)=0] \\ &= \sum_{k,l} y_3^{k} z^{l} \Pr[M(t)=k, D(t)=l | M(\tau)=1, D(\tau)=0] \end{aligned} \tag{13}$$

$$\begin{aligned} \Phi_{P}(y_2,y_3,z;\tau,t) &= E[y_2^{P(t)} y_3^{M(t)} z^{D(t)} | P(\tau)=1, M(\tau)=0, D(\tau)=0] \\ &= \sum_{j,k,l} y_2^{j} y_3^{k} z^{l} \Pr[P(t)=j, M(t)=k, D(t)=l | P(\tau)=1, M(\tau)=0, D(\tau)=0] \end{aligned} \tag{14}$$

$$\begin{aligned} \Phi_{P^{*}}(y_1,y_2,y_3,z;\tau,t) &= E[y_1^{P^{*}(t)} y_2^{P(t)} y_3^{M(t)} z^{D(t)} | P^{*}(\tau)=1, P(\tau)=0, M(\tau)=0, D(\tau)=0] \\ &= \sum_{i,j,k,l} y_1^{i} y_2^{j} y_3^{k} z^{l} \Pr[P^{*}(t)=i, P(t)=j, M(t)=k, D(t)=l | P^{*}(\tau)=1, P(\tau)=0, M(\tau)=0, D(\tau)=0] \end{aligned} \tag{15}$$

$$\Phi_{BE}(y_{BE},y_1,y_2,y_3,z;\tau,t) = E[y_{BE}^{BE(t)} y_1^{P^{*}(t)} y_2^{P(t)} y_3^{M(t)} z^{D(t)} | BE(\tau)=1, P^{*}(\tau)=0, P(\tau)=0, M(\tau)=0, D(\tau)=0] \tag{16}$$

$$= \sum_{i,j,k,l,n} y_{BE}^i y_1^j y_2^j y_3^l z^n \Pr[BE(t)=i, P^*(t)=j, P(t)=k, M(t)=l, D(t)=n | BE(\tau)=1, P^*(\tau)=0, P(\tau)=0, M(\tau)=0, D(\tau)=0]$$

The generating functions satisfy the following Kolmogorov backward equations

$$\frac{\partial \Phi_M(y_3, z; \tau, t)}{\partial \tau} = -\alpha_M \Phi_M^2(y_3, z; \tau, t) - \beta_M - z\rho \Phi_M(y_3, z; \tau, t) + [\alpha_M + \beta_M + \rho]\Phi_M(y_3, z; \tau, t) \quad (17)$$

$$\frac{\partial \Phi_P(y_2, y_3, z; \tau, t)}{\partial \tau} = -\alpha_P \Phi_P^2(y_2, y_3, z; \tau, t) - \beta_P + [\alpha_P + \beta_P + \mu_2]\Phi_P(y_2, y_3, z; \tau, t) - \mu_2 \Phi_P(y_2, y_3, z; \tau, t)\Phi_M(y_3, z; \tau, t) \quad (18)$$

$$\frac{\partial \Phi_{P*}(y_1, y_2, y_3, z; \tau, t)}{\partial \tau} = -\mu_1 \Phi_{P*}(y_1, y_2, y_3, z; \tau, t)[\Phi_P(y_2, y_3, z; \tau, t) - 1] \quad (19)$$

$$\frac{\partial \Phi_{BE}(y_{BE}, y_1, y_2, y_3, z; \tau, t)}{\partial \tau} = -\mu_0 X \Phi_{BE}(y_{BE}, y_1, y_2, y_3, z; \tau, t)[\Phi_{P*}(y_1, y_2, y_3, z; \tau, t) - 1] \quad (20)$$

$$\frac{\partial \Psi(y_{BE}, y_1, y_2, y_3, z; \tau, t)}{\partial \tau} = \nu(\tau)[\Psi(y_{BE}, y_1, y_2, y_3, z; \tau, t) - \Phi_{BE}(y_{BE}, y_1, y_2, y_3, z; \tau, t)] \quad (21)$$

To connect the cellular level description to the population level, we first solve for the overall survival function (for EAC cancer detection), starting at time 0, which in our notation is

$$S_{EAC}(t) = 1 - P_{EAC}(t) = \Pr[D(t) = 0 | BE(0) = 0, P^*(0) = 0, P(0) = 0, M(0) = 0, D(0) = 0] = \Psi(1, 1, 1, 1, 0; 0, t)$$

where $P_{EAC}(t)$ is the probability of a cancer detection at time t,

$$P_{EAC}(t) = \Pr[D(t) = 1 | BE(0) = 0, P^*(0) = 0, P(0) = 0, M(0) = 0, D(0) = 0]$$

We will here denote $\Phi_M(1, 0; \tau, t) \equiv \Phi_M(\tau, t)$, $\Phi_P(1, 1, 0; \tau, t) \equiv \Phi_P(\tau, t)$, $\Phi_{P*}(1, 1, 1, 0; \tau, t) \equiv \Phi_{P*}(\tau, t)$, $\Phi_{BE}(1, 1, 1, 1, 0; \tau, t) \equiv \Phi_{BE}(\tau, t)$, and $\Psi(1, 1, 1, 1, 0; \tau, t) \equiv \Psi(\tau, t)$. A dot designates a first derivative with respect to t. The hazard function, i.e., the rate at which cancer is detected in individuals who have not been diagnosed before, is given by

$$h_{EAC}(t) = -\frac{\dot{S}_{EAC}(t)}{S_{EAC}(t)} = -\frac{\dot{\Psi}(0, t)}{\Psi(0, t)} = -\frac{d}{dt}\ln[\Psi(0, t)] \quad (22)$$

For fixed t, this boundary value system of coupled PDEs can be converted into an initial value problem (IVP) with the change of variables $u = t - \tau$, where u is the "running" time. This redefinition and equations hereafter follow the method used by Crump et al. [8]. Define the following variables for the new IVP: $Y_1(u, t) = \Phi_M(\tau, t), Y_2(u, t) = \dot{\Phi}_M(\tau, t), Y_3(u, t) = \Phi_P(\tau, t), Y_4(u, t) = \dot{\Phi}_P(\tau, t), Y_5(u, t) = \Phi_{P*}(\tau, t), Y_6(u, t) = \dot{\Phi}_{P*}(\tau, t), Y_7(u, t) = \Phi_{BE}(\tau, t), Y_8(u, t) = \dot{\Phi}_{BE}(\tau, t), Y_9(u, t) = \Psi(\tau, t), Y_{10}(u, t) = \dot{\Psi}(\tau, t)$ with corresponding initial conditions $Y_1(0, t) = Y_3(0, t) = Y_5(0, t) = Y_7(0, t) = Y_9(0, t) = 1, Y_4(0, t) = Y_6(0, t) = Y_8(0, t) = Y_{10}(0, t) = 0$, and $Y_2(0, t) = -\rho$. Then the

equations to solve for our IVP are the following

$$\frac{dY_1(u,t)}{du} = \beta_M - (\alpha_M + \beta_M + \rho)Y_1(u,t) + \alpha_M Y_1^2(u,t) \tag{23}$$

$$\frac{dY_2(u,t)}{du} = 2\alpha_M Y_1(u,t)Y_2(u,t) - (\alpha_M + \beta_M + \rho)Y_2(u,t) \tag{24}$$

$$\frac{dY_3(u,t)}{du} = \beta_P + \mu_2 Y_1(u,t)Y_3(u,t) - (\alpha_P + \beta_P + \mu_2)Y_3(u,t) + \alpha_P Y_3^2(u,t) \tag{25}$$

$$\frac{dY_4(u,t)}{du} = 2\alpha_P Y_3(u,t)Y_4(u,t) + \mu_2(Y_4(u,t)Y_1(u,t) + Y_3(u,t)Y_2(u,t)) - (\alpha_P + \beta_P + \mu_2)Y_4(u,t) \tag{26}$$

$$\frac{dY_5(u,t)}{du} = \mu_1 Y_5(u,t)(Y_3(u,t) - 1) \tag{27}$$

$$\frac{dY_6(u,t)}{du} = \mu_1(Y_6(u,t)Y_3(u,t) - Y_6(u,t) + Y_5(u,t)Y_4(u,t)) \tag{28}$$

$$\frac{dY_7(u,t)}{du} = \mu_0 X Y_7(u,t)(Y_5(u,t) - 1) \tag{29}$$

$$\frac{dY_8(u,t)}{du} = \mu_0 X(Y_8(u,t)Y_5(u,t) - Y_8(u,t) + Y_7(u,t)Y_6(u,t)) \tag{30}$$

$$\frac{dY_9(u,t)}{du} = \nu(u)(Y_7(u,t) - Y_9(u,t)) \tag{31}$$

$$\frac{dY_{10}(u,t)}{du} = \nu(u)(Y_{10}(u,t) - Y_8(u,t)). \tag{32}$$

These 10 coupled ODEs can be solved numerically to obtain the desired survival function conditional on time of BE onset from Eq. (12),

$$S_{MSCE}(t) = Y_7(t,t). \tag{33}$$

References

1. Gelfand AE, Smith AF. Sampling-based approaches to calculating marginal densities. Journal of the American statistical association. 1990;85(410):398–409.

2. Surveillance, Epidemiology, and End Results (SEER) Program (www.seer.cancer.gov) SEER*Stat Database: Incidence - SEER 9 Regs Research Data, Nov 2013 Sub (1973-2011) Katrina/Rita Population Adjustment - Linked To County Attributes - Total U.S., 1969-2012 Counties, National Cancer Institute, DCCPS, Surveillance Research Program, Surveillance Systems Branch, released April 2014, based on the November 2013 submission.;.

3. Kong CY, Kroep S, Curtius K, Hazelton WD, Jeon J, Meza R, et al. Exploring the Recent Trend in Esophageal Adenocarcinoma Incidence and Mortality Using Comparative Simulation Modeling. Cancer Epidemiol Biomarkers Prev. 2014;23(6):997–1006.

4. Curtius K, Hazelton W, Jeon J, Luebeck E. A Multiscale Model Evaluates Screening for Neoplasia in Barrett's Esophagus. PLoS Comput Biol. 2015;11(5):e1004272.

5. Moolgavkar S, Dewanji A, Venzon D. A Stochastic Two-Stage Model for Cancer Risk Assessment. II. The Number and Size of Premalignant Clones. The Hazard Function and the Probability of Tumor. Risk Anal. 1988;8(3):383–392.

6. Little M. Are two mutations sufficient to cause cancer? Some generalizations of the two-mutation model of carcinogenesis of Moolgavkar, Venzon, and Knudson, and of the multistage model of Armitage and Doll. Biometrics. 1995;51:1278–1291.

7. Luebeck E, Moolgavkar S. Multistage carcinogenesis and the incidence of colorectal cancer. Proc Natl Acad Sci U S A. 2002;99:15095–15100.

8. Crump C, Subramaniam R, Van Landingham C. A Numerical Solution to the Nonhomogeneous Two-Stage MVK Model of Cancer. Risk Anal. 2005;25:921–926.
