# Supplementary figures and images for: A Molecular Clock Infers Heterogeneous Tissue Age Among Patients with Barrett’s Esophagus
Source: PLoS Comput Biol. 2016 May 11;12(5):e1004919. doi: 10.1371/journal.pcbi.1004919 (PMC4864310; doi:10.1371/journal.pcbi.1004919)

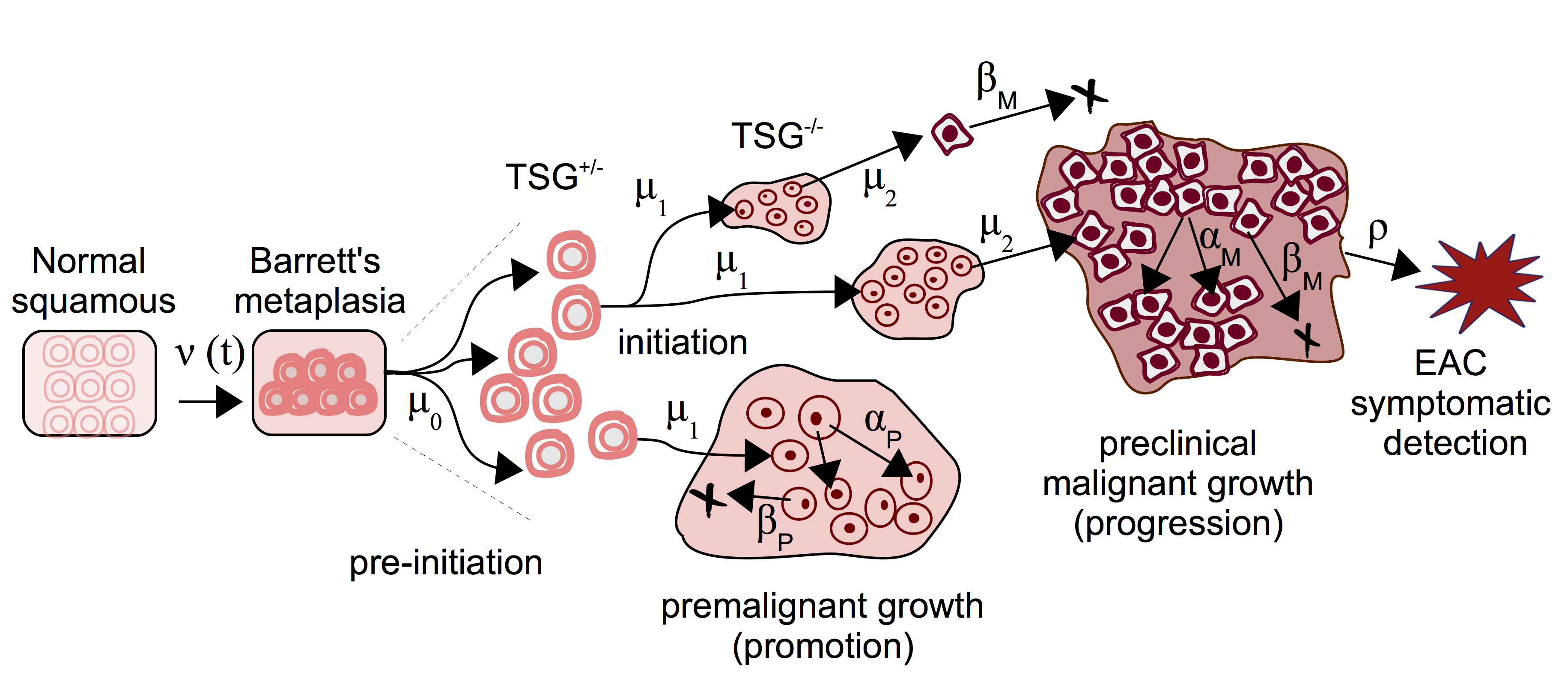

Supplement: S1 Fig — Normal squamous epithelium may transform to BE with an exponentially distributed onset time with rate ν(t), followed by a ‘two-hit’ tumor initiation process with Poisson initiation rates μ0, μ1, which leads to the stochastic appearance of premalignant progenitor cells in the tissue. Premalignant cells undergo a first clonal expansion described by a birth-death-migration process with cell division rate αP, cell death-or-differentiation rate βP, and malignant transformation rate μ2. Malignant cells, in turn, undergo a second clonal expansion by a birth-death-detection process with cell division and death rates αM and βM, respectively, allowing for stochastic growth and possibly extinction of the malignant tumor. Clinical detection occurs through a size-based detection process with parameter ρ. TSG, tumor suppressor gene [16]. (TIFF) [file pcbi.1004919.s002.tiff]

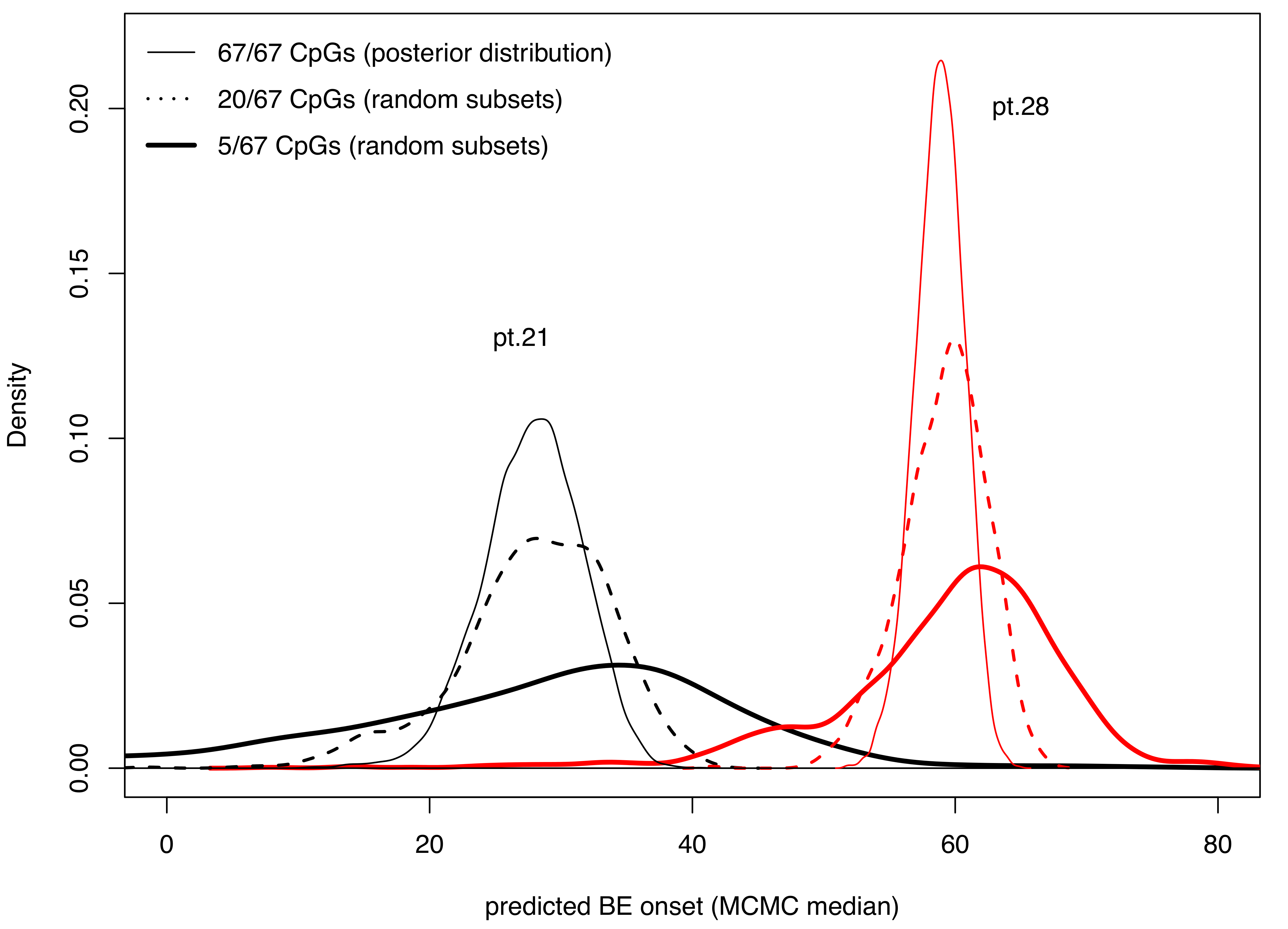

Supplement: S2 Fig — Comparison of the posterior distributions of BE onsets for two 84 year old BE patients (pt. 21 and pt. 28) in study D2 using the identified set of 67 BE clock CpGs (thin solid line). To test the relative robustness of the estimated mean BE onsets, we also generated random subsamples (without replacement) of size 5 and 20 from the 67 clock CpGs. Shown are the distributions of the median BE onset estimates using MCMC (5K cycles) for n = 5 CpGs (thick solid lines) and n = 20 CpGs (dashed line). (TIF) [file pcbi.1004919.s003.tif]

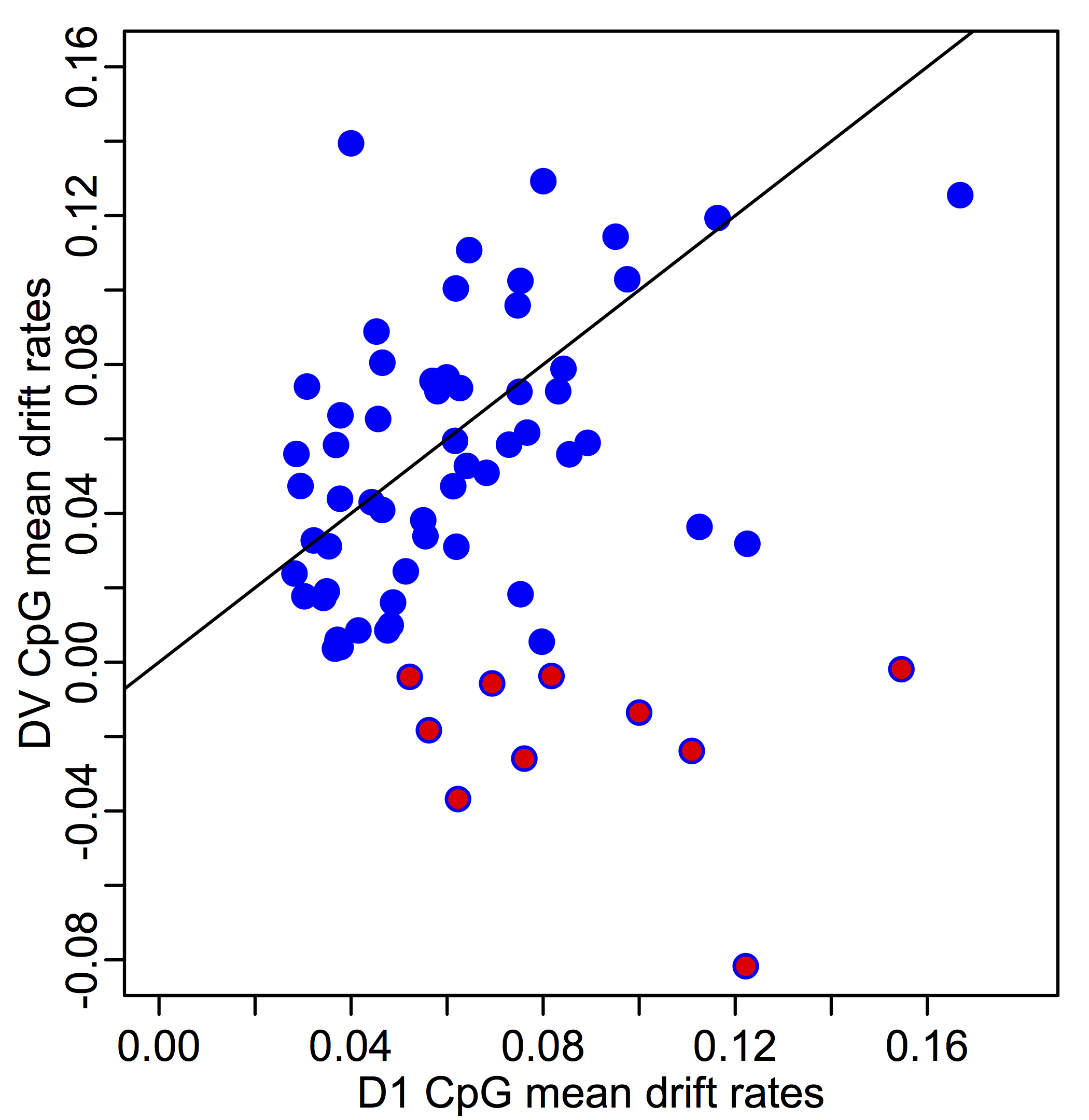

Supplement: S3 Fig — Between the entire sets D1 and DV, we see relatively low correlation for mean marker-specific drift rates calculated via linear regression (see Methods). However, this plot does suggest that there are outliers (negative bj rates in DV colored in red) that hide an interesting correlation. Rather than homogenous drift, the correlation between longitudinal drift rates in D1 and DV (with outliers removed, corr = 0.45, p-value < 0.05) suggests the presence of heterogeneity in marker-specific drift rates. Ultimately, there was minimal effect conferred on posterior parameter estimates due to “winner’s curse” bias inherent in the D1 drift rates calculated during BE clock marker selection versus validation DV drift rates when used as two candidate priors in the MCMC (see S1 Text). (TIFF) [file pcbi.1004919.s004.tiff]

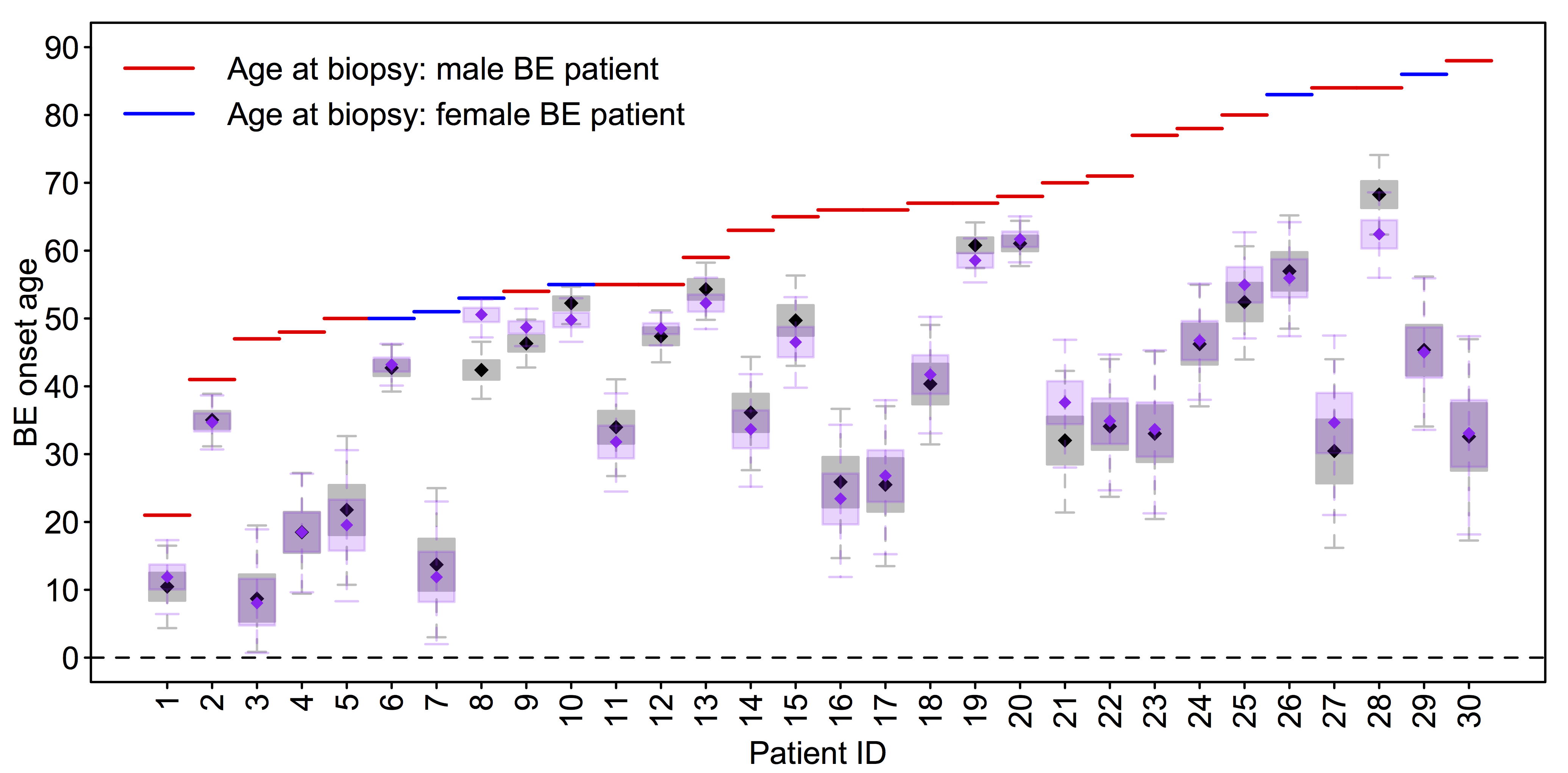

Supplement: S4 Fig — For the unmasked (grey boxplots) and masked (purple boxplots) implementations of inferring BE onset ages, we found that using an imputation of the intercept and drift rates of SQ tissue values across the D2 patients rather than exact matched SQ values is a robust approach (see S1 Text for details). Specifically, the correlation of median estimates between the two methods was .98, and the root-mean-square error between onset ages was 0.08 years. (TIFF) [file pcbi.1004919.s005.tiff]

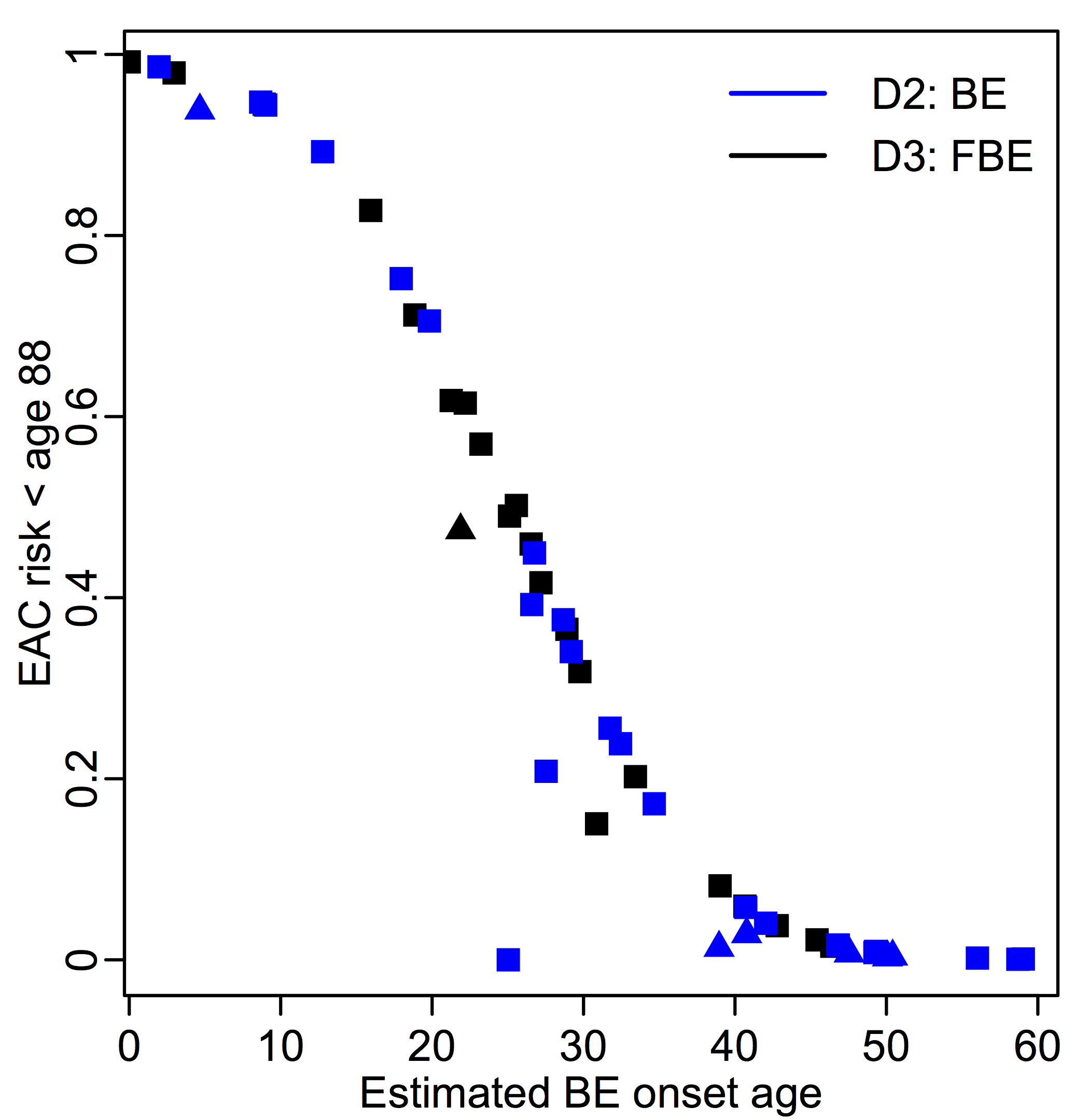

Supplement: S5 Fig — Across patients in data sets D2 (blue points) and D3 (black points), there is high correlation between the median MCMC posterior estimates for BE onset age and the corresponding EAC risk before age 88 as predicted by the multistage clonal expansion model (S1 Fig) that utilizes BE onset as an input. The stochastic model predicts that risk increases exponentially with earlier BE onset ages for patients of similar age. Square points designate males, triangle points designate females. (TIFF) [file pcbi.1004919.s006.tiff]
